# Pneumonia surveillance with culture-independent metatranscriptomics in HIV-positive adults in Uganda: a cross-sectional study

**DOI:** 10.1016/S2666-5247(21)00357-8

**Published:** 2022-03-25

**Authors:** Natasha Spottiswoode, Joshua D Bloomstein, Saharai Caldera, Abdul Sessolo, Kathryn McCauley, Patrick Byanyima, Josephine Zawedde, Katrina Kalantar, Sylvia Kaswabuli, Rachel L Rutishauser, Monica K Lieng, J Lucian Davis, Julia Moore, Amanda Jan, Shoko Iwai, Meera Shenoy, Ingvar Sanyu, Joseph L DeRisi, Susan V Lynch, William Worodria, Laurence Huang, Charles R Langelier

**Affiliations:** Department of Medicine, University of California, San Francisco, San Francisco, CA, USA; Division of Infectious Diseases, University of California, San Francisco, San Francisco, CA, USA; Department of Medicine, University of California Davis School of Medicine, Sacramento, CA, USA; Division of Infectious Diseases, University of California, San Francisco, San Francisco, CA, USA; Chan Zuckerberg Biohub, San Francisco, CA, USA; Infectious Disease Platform, Makerere University, Kampala, Uganda; Division of Gastroenterology, University of California, San Francisco, San Francisco, CA, USA; Infectious Disease Platform, Makerere University, Kampala, Uganda; Infectious Disease Platform, Makerere University, Kampala, Uganda; Chan Zuckerberg Initiative, Redwood City, CA, USA; Infectious Disease Platform, Makerere University, Kampala, Uganda; Department of Medicine, University of California, San Francisco, San Francisco, CA, USA; Division of Experimental Medicine, University of California, San Francisco, San Francisco, CA, USA; Department of Medicine, University of California Davis School of Medicine, Sacramento, CA, USA; Department of Epidemiology of Microbial Diseases, Yale School of Public Health and Pulmonary, Critical Care, and Sleep Medicine, Yale School of Medicine, New Haven, CT, USA; Division of Pulmonary and Critical Care Medicine, University of California, San Francisco, San Francisco, CA, USA; Division of HIV, Infectious Diseases, and Global Medicine, University of California, San Francisco, San Francisco, CA, USA; Department of Epidemiology of Microbial Diseases, Yale School of Public Health and Pulmonary, Critical Care, and Sleep Medicine, Yale School of Medicine, New Haven, CT, USA; Division of Gastroenterology, University of California, San Francisco, San Francisco, CA, USA; Division of Gastroenterology, University of California, San Francisco, San Francisco, CA, USA; Infectious Disease Platform, Makerere University, Kampala, Uganda; Department of Biochemistry, University of California, San Francisco, San Francisco, CA, USA; Chan Zuckerberg Biohub, San Francisco, CA, USA; Division of Gastroenterology, University of California, San Francisco, San Francisco, CA, USA; Infectious Disease Platform, Makerere University, Kampala, Uganda; Division of Pulmonary and Critical Care Medicine, University of California, San Francisco, San Francisco, CA, USA; Division of HIV, Infectious Diseases, and Global Medicine, University of California, San Francisco, San Francisco, CA, USA; Department of Medicine, University of California, San Francisco, San Francisco, CA, USA; Division of Infectious Diseases, University of California, San Francisco, San Francisco, CA, USA; Chan Zuckerberg Biohub, San Francisco, CA, USA

## Abstract

**Background:**

Pneumonia is a leading cause of death worldwide and is a major health-care challenge in people living with HIV. Despite this, the causes of pneumonia in this population remain poorly understood. We aimed to assess the feasibility of metatranscriptomics for epidemiological surveillance of pneumonia in patients with HIV in Uganda.

**Methods:**

We performed a retrospective observational study in patients with HIV who were admitted to Mulago Hospital, Kampala, Uganda between Oct 1, 2009, and Dec 31, 2011. Inclusion criteria were age 18 years or older, HIV-positivity, and clinically diagnosed pneumonia. Exclusion criteria were contraindication to bronchoscopy or an existing diagnosis of tuberculosis. Bronchoalveolar lavage fluid was collected within 72 h of admission and a combination of RNA sequencing and *Mycobacterium tuberculosis* culture plus PCR were performed. The primary outcome was detection of an established or possible respiratory pathogen in the total study population.

**Findings:**

We consecutively enrolled 217 patients during the study period. A potential microbial cause for pneumonia was identified in 211 (97%) patients. At least one microorganism of established respiratory pathogenicity was identified in 113 (52%) patients, and a microbe of possible pathogenicity was identified in an additional 98 (45%). *M tuberculosis* was the most commonly identified established pathogen (35 [16%] patients; in whom bacterial or viral co-infections were identified in 13 [37%]). *Streptococcus mitis*, although not previously reported as a cause of pneumonia in patients with HIV, was the most commonly identified bacterial organism (37 [17%] patients). *Haemophilus influenzae* was the most commonly identified established bacterial pathogen (20 [9%] patients). *Pneumocystis jirovecii* was only identified in patients with a CD4 count of less than 200 cells per mL.

**Interpretation:**

We show the feasibility of using metatranscriptomics for epidemiologic surveillance of pneumonia by describing the spectrum of respiratory pathogens in adults with HIV in Uganda. Applying these methods to a contemporary cohort could enable broad assessment of changes in pneumonia aetiology following the emergence of SARS-CoV-2.

**Funding:**

US National Institutes of Health, Chan Zuckerberg Biohub.

## Introduction

Sub-Saharan Africa has a disproportionate burden of the global HIV pandemic and, in Uganda alone, an estimated 21 000 people died of HIV-related causes in 2019.^1^ People living with HIV have a higher risk of developing pneumonia and experience more severe disease than immunocompetent individuals.^2^ Lower respiratory tract infections are the leading cause of death among people with HIV in Uganda and other low-resource countries, and bacterial pneumonia is associated with mortality rates of greater than 20%, even with antiretroviral and antibiotic treatments.^3^

Understanding the causes of pneumonia across geographical regions is essential for informing empirical treatment guidelines, tracking emerging pathogens, and guiding vaccination efforts in regions with limited clinical diagnostic infrastructure. Pneumonia diagnoses in people living with HIV pose an additional diagnostic challenge as these patients are at risk from a broad array of respiratory pathogens.^4^ At present, the causes of pneumonia in adults with HIV residing in Uganda and other African nations remain incompletely understood. This is largely due to the low sensitivity of using conventional microbial culture in patients who have already received empirical antibiotic treatment,^5^ and the intrinsic limitations of PCR assays to detect uncommon or emerging pathogens.^6,7^ In a landmark study, traditional diagnostic methodologies were shown to lead to identification of a causative pathogen in only 40% of pneumonia cases.^8^

Metatranscriptomic RNA sequencing provides an advantageous and complementary approach to traditional infectious disease surveillance methods and is particularly well suited for regions with limited clinical microbiological testing capacity. By affording culture-independent broad-range assessment of bacterial, viral, mycobacterial, fungal, and parasitic species from a single clinical sample, metatranscriptomic RNA sequencing can reduce the need for multiple types of testing platforms (eg, culture, serology, or pathogen-specific PCRs).^9-11^ A previous study has highlighted the utility of metatranscriptomics for the diagnosis of complicated lower respiratory tract infections, and bioinformatics advancements now enable detection of respiratory pathogens among the ubiquitous and complex background of commensal airway microbiota.^9^ Open-access cloud-based bioinformatics pipelines have democratised the computationally intense RNA sequencing data analysis, increasing availability and applicability in low-resource settings.^12^

To our knowledge, no studies to date have used metatranscriptomic RNA sequencing for lower respiratory tract infection surveillance in sub-Saharan Africa, and few have examined the causes of pneumonia using this approach in people living with HIV. We aimed to assess the feasibility of metatranscriptomics for epidemiological surveillance of pneumonia in patients with HIV in Uganda.

## Methods

### Study design and participants

We performed a retrospective observational study in patients who were admitted to Mulago Hospital, Kampala, Uganda between Oct 1, 2009, and Dec 31, 2011. Adults (age ≥18 years) with HIV-positivity and clinically diagnosed pneumonia, defined as the presence of cough and a chest radiograph consistent with pneumonia, as well as two negative smears for acid-fast bacilli, were enrolled following written informed consent via the prospective Mulago Inpatient Non-invasive Diagnosis (MIND)-International HIV-associated Opportunistic Pneumonias (IHOP) cohort study.^13,14^ Exclusion criteria were contraindication to bronchoscopy, or an existing diagnosis of tuberculosis or positive acid-fast bacilli smear (appendix p 2). Mulago Hospital is a tertiary care referral centre. The study was approved by the Mulago Institutional Review Board (2006-0174) and the University of California, San Francisco Institutional Review Board (10-02633).

### Procedures

Baseline clinical information was collected, including age, sex, CD4 cell count, and background antiretrovirals and antibiotics. Patients provided two sputum samples for acid-fast bacilli smear examination and cultures to diagnose pulmonary *Mycobacterium tuberculosis*. Patients with a negative acid-fast bacilli smear had bronchoscopy with bronchoalveolar lavage for clinical diagnosis (appendix p 3). *M tuberculosis* testing was carried out using acid-fast bacilli culture on expectorated or induced sputum and on bronchoalveolar lavage. In addition, sputum PCR for *M tuberculosis* and rifampicin resistance, using the GeneXpert MTB/RIF assay, was performed in patients for whom the assay was available during their admission. *Pneumocystis jirovecii* was detected by using a combination of Giemsa staining and RNA sequencing. Data from the cohort in this study have been previously reported in studies that performed analyses of pulmonary bacterial pathogens via 16S rRNA gene sequencing.^13,14^

RNA extraction from bronchoalveolar lavage was done as described in previous studies,^13,14^ and was used for library preparation and Illumina sequencing as described in the appendix (p 3).

The open-source IDseq pipeline was used to detect respiratory microbes from RNA sequencing data (appendix p 4).^15^ Antimicrobial resistance genes were also screened in the metagenomic dataset (appendix p 7). To identify established pneumonia pathogens and distinguish them from microbes of possible pathogenicity and commensal microbiota, we used a previously validated rules-based model (appendix pp 5-6).^9^ The rules-based model incorporates previous findings which showed that microbial communities in patients with lower respiratory tract infections are typically characterised by one or more dominant pathogens present in high abundance (appendix p 10).^9^ Specifically, the rules-based model ranks microbes present in a sample by descending abundance (ie, number of taxonomic alignments) and identifies the greatest difference in abundance between any two sequential taxa. This split point divides the taxa into high abundance and low abundance and defines identification. On the basis of this principle, the rules-based model identifies the subset of bacteria and fungi with the greatest relative abundance in each sample, which consist of single microbes in cases of a dominant pathogen. In cases of co-infections, the rules-based model can identify several microbes present at similar disproportionately high abundance compared with the rest of the lung microbiota. Because viral load varies over the course of lower respiratory tract infection, the rules-based model identifies all viruses with greater than a background threshold of 0·1 viral reads per million sequenced. RNA sequencing data was used as input for the rules-based model to identify organisms, except for *M tuberculosis*, for which culture and PCR were performed.

If the microbe identified by the rules-based model was present within a reference index of established respiratory pathogens, derived from landmark surveillance studies and clinical guidelines (appendix pp 5-6, 13-14),^8,16-18^ it was selected as an established pathogen by the model. Viral, bacterial, and fungal taxa identified by the rules-based model but not included on the reference list of established respiratory pathogens were considered possible respiratory pathogens. Established pathogens were subdivided into bacteria, viruses, mycobacteria, and fungi, and were further analysed by group.

The primary study outcome was detection of an established or possible respiratory pathogen in the total study population. Secondary outcomes were associations between pathogens and CD4 cell count and 70-day mortality, and associations between trimethoprim–sulfamethoxazole use and *P jirovecii* pneumonia and trimethoprim–sulfamethoxazole resistance genes in the total study population.

### Statistical analysis

Mann-Whitney *U* tests were used to compare CD4 cell counts with respect to binary outcomes (70-day mortality). ANOVA was used to compare CD4 cell counts across four patient groups defined by pathogen category (established and possible pathogens, established pathogens only, possible pathogens only, and no pathogen detected). Fisher’s exact test was used to test for association of pathogen with the categorical variables of CD4 count (≥200 *vs* <200 cells per mL) and mortality. Fisher’s exact test was also used to assess the relationship between trimethoprim–sulfamethoxazole prophylaxis and *P jirovecii* infection or trimethoprim–sulfamethoxazole resistance gene detection. Significance was defined as a p value of less than 0·05. Adjusted p values were calculated with the Benjamini–Hochberg method and are reported in the appendix (pp 25-26). Analyses were performed with R (version 4.0.2) and GraphPad Prism (version 9.2.0).

### Role of the funding source

The funder of the study had no role in study design, data collection, data analysis, data interpretation, or writing of the report.

## Results

**Figure 1:**
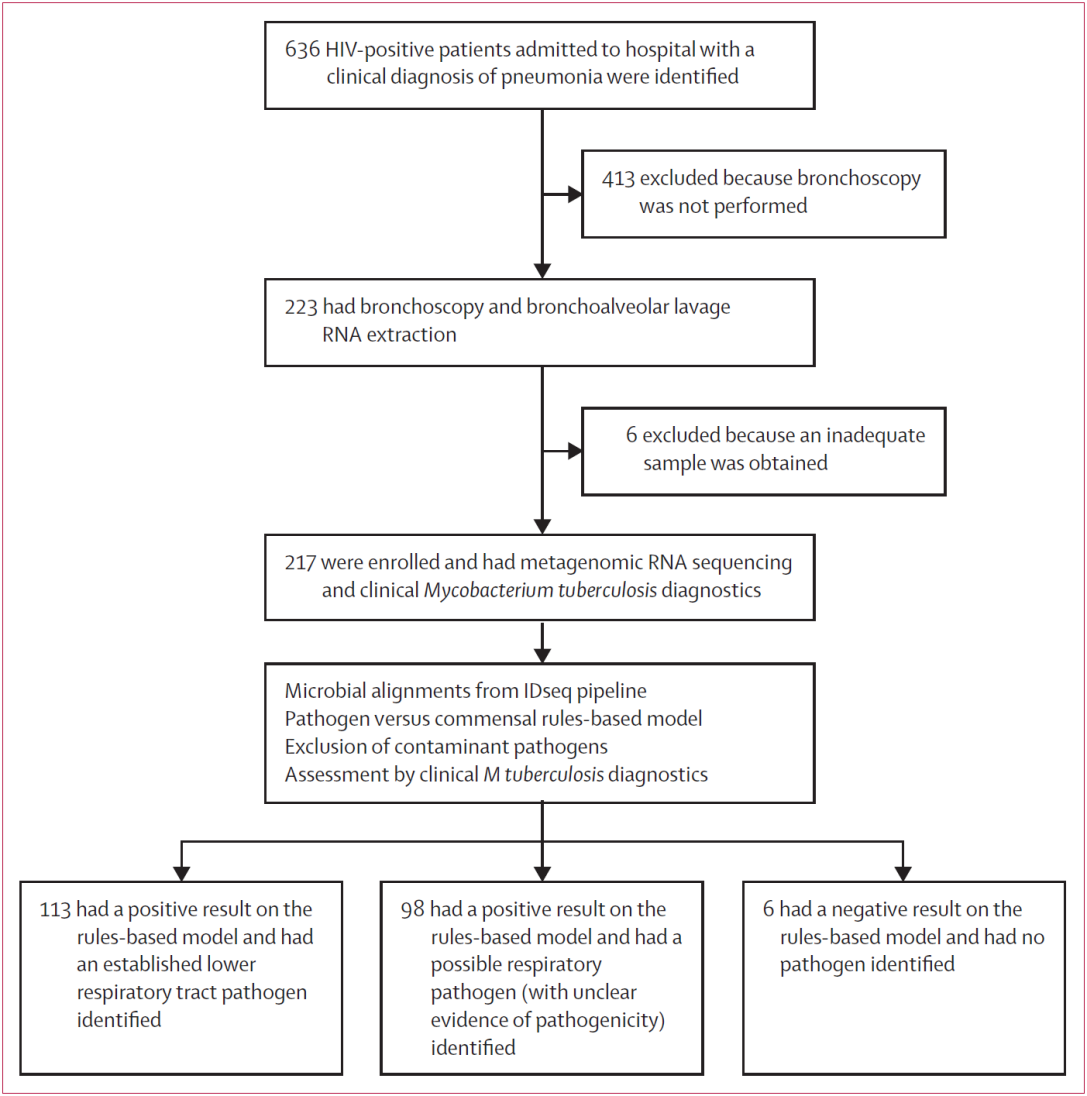
Study profile

We consecutively enrolled 217 patients during the study period (table, figure 1, appendix p 34), as described previously.^13,14^ At the time of admission to hospital, 164 (76%) of 217 patients had a CD4 count of less than 200 cells per mL, 48 (22%) were taking antiretroviral therapy, and 122 (56%) had been receiving *P jirovecii* pneumonia prophylaxis. 183 (84%) of 217 patients were receiving empirical antimicrobial therapy. 147 (68%) patients received in-hospital trimethoprim–sulfamethoxazole for empirical treatment of *P jirovecii* pneumonia (table). Mortality data at 70 days after enrolment were collected for 194 (89%) of 217 patients, 47 (24%) of whom had died. Sputum PCR was available for and performed in 137 (63%) of 217 patients.

**Figure 2:**
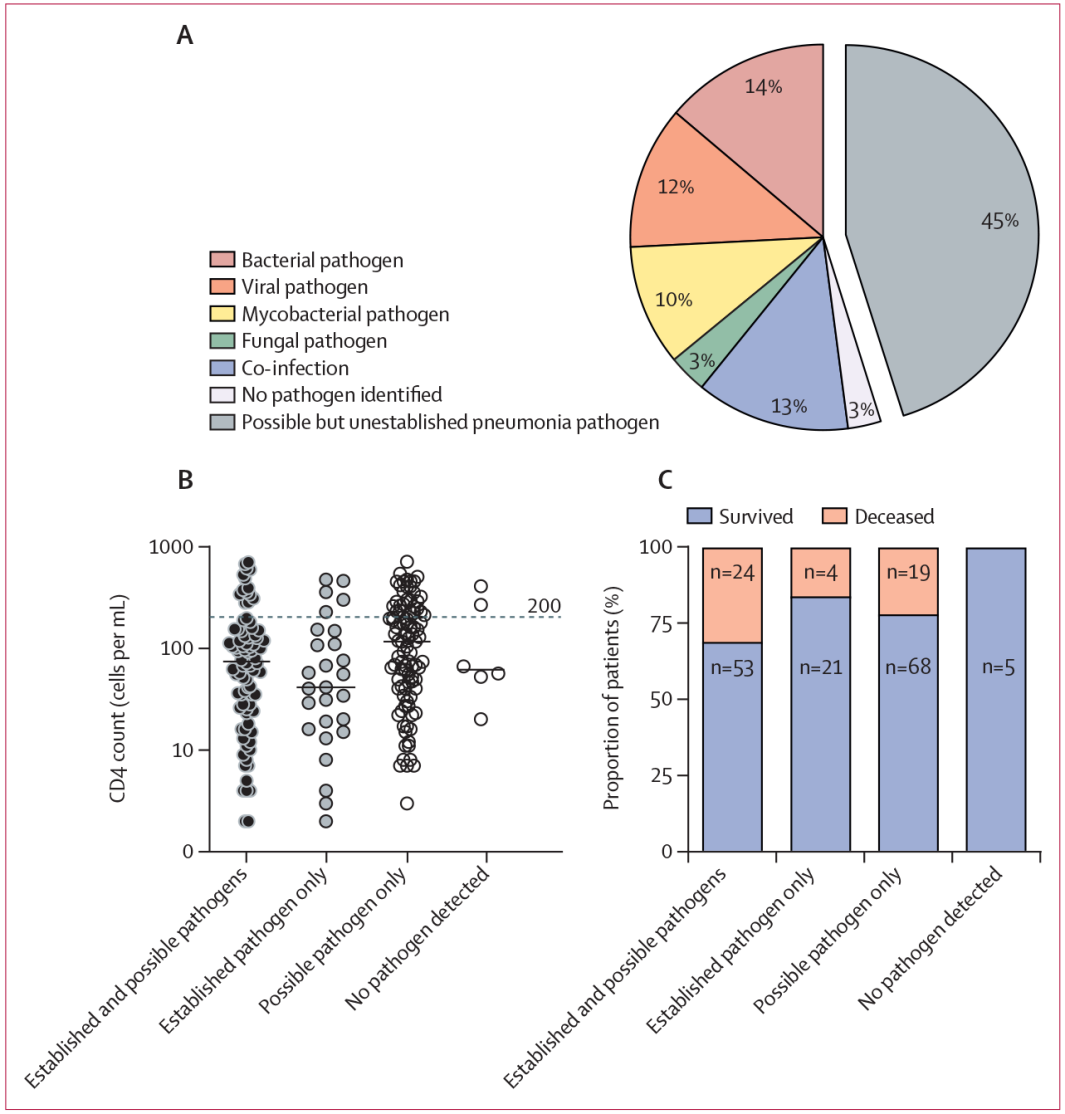
Pneumonia pathogens identified in the study cohort (A) Breakdown by pathogen type in 217 patients; two or more pathogen types are grouped regardless of the pathogens identified (blue section). Grey section indicates atypical pathogens with possible, but not yet clearly established, lower respiratory tract pathogenicity. (B) Baseline CD4 cell counts compared between patients with both established and possible pathogens, only established pathogens, only possible pathogens, and no pathogens detected. Each dot represents a single patient, and medians are depicted by horizontal bars. There was no significant difference between groups calculated by ANOVA. (C) Mortality differences at day 70 after enrolment between patients with both established and possible pathogens, only established pathogens, only possible pathogens, and no pathogens detected. No group had a significant association with mortality calculated by Fisher’s exact test. Patients without survival data available were excluded from this analysis.

The combination of RNA sequencing and *M tuberculosis* clinical diagnostics identified at least one organism of possible pathogenicity for pneumonia in 211 (97%) of 217 patients (appendix p 15). Six patients (3%) had no identified pathogen. After filtering the rules-based model predictions using the reference list of pneumonia pathogens, an established lower respiratory pathogen was identified in 113 (52%) of 217 patients (figure 2, appendix p 17). Possible respiratory pathogens, representing microbes with limited literature evidence for respiratory pathogenicity in published case series or scientific reports, were identified in 98 (45%) patients (figure 2, appendix p 19).

Of 113 patients with established lower respiratory tract pathogens, 28 (25%) had pathogens representing more than one group (bacteria, viruses, mycobacteria, or fungi). Viral pathogens alone were detected in 26 (23%) patients, non-mycobacterial bacterial pathogens alone in 30 (27%), mycobacterial pathogens alone in 22 (19%), and fungal pathogens alone in seven (6%; figure 2, appendix p 15). There was no significant difference in 70-day mortality between patients with an established lower respiratory tract pathogen and those without (p=0·32).

The most commonly identified established pathogens were *M tuberculosis,* human rhinovirus, *Haemophilus influenzae, P jirovecii, Pseudomonas aeruginosa, Streptococcus pneumoniae, Klebsiella pneumoniae*, influenza virus, and human metapneumovirus. Of 113 patients with established pathogens, 30 (27%) had more than one established pathogen (appendix pp 11, 15).

Of the established bacterial pathogens, *H influenzae* was the most commonly identified respiratory bacterial pathogen (20 [9%] of 217 patients), followed by *P aeruginosa* (12 [6%]), *S pneumoniae* (seven [3%]), and *K pneumoniae* (seven [3%]; appendix p 17). Among the established viral pathogens, human rhinoviruses were the most common (34 [16%] patients), followed by influenza A virus (six [3%]) and human metapneumovirus (four [2%]).

*M tuberculosis* was identified in 35 (16%) of 217 patients by a combination of acid-fast bacilli culture, GeneXpert MTB/RIF assay, and RNA sequencing (appendix p 16). Among patients with *M tuberculosis*, 13 (37%) of 35 had at least one additional established pathogen identified, the most common of which was human rhinovirus, followed by *P aeruginosa* and *H influenzae* (appendix p 17). With regard to established fungal pathogens, *P jirovecii* was detected with a combination of RNA sequencing and Giemsa staining in 12 (6%) of 217 patients. One (<1%) patient was found to have *Histoplasma capsulatum* (appendix p 17).

Microbes with incompletely established evidence of respiratory tract pathogenicity (as defined in previous work)^9^ were identified in 183 (84%) of 217 patients. Bacteria included *Veillonella* spp (59 [27%] patients), *Streptococcus* spp (excluding *S pneumoniae*; 47 [22%]), *Pseudomonas* spp (excluding *P aeruginosa*; 28 [13%]), *Prevotella* spp (18 [8%]), and *Actinomyces* spp (ten [5%]; appendix pp 11, 19). Among patients with *Streptococcus* spp (excluding *S pneumoniae*), *Streptococcus mitis* was the most common pathogen (36 [77%] of 47 patients) and among those with *Pseudomonas* spp, *Pseudomonas putida* was the most common (14 [50%] of 28; appendix p 11). Other potential pathogens included *Enterobacter kobei*, *Rhodococcus hoagi*, *Burkholderia ambifara*, *Acinetobacter schindleri*, and *Tropheryma whipplei* (appendix p 19).

With respect to potential viral pneumonia pathogens, rubella virus was detected in one (<1%) of 217 patients and many patients were found to have viruses of unclear respiratory pathogenicity. These included HIV-1 (151 [70%] patients), human herpes virus 4 (Epstein-Barr virus; 69 [32%]), Anelloviridae (including torque teno viruses; 49 [23%]), human herpes virus 5 (cytomegalovirus; 24 [11%]), human herpes virus 8 (Kaposi sarcoma-associated herpes virus; 18 [8%]), human alpha herpes virus 1 (five [2%]), aichivirus (three [1%]), hepatitis B virus (three [1%]), pegivirus (two [1%]), and norovirus (one [<1%]).

**Figure 3:**
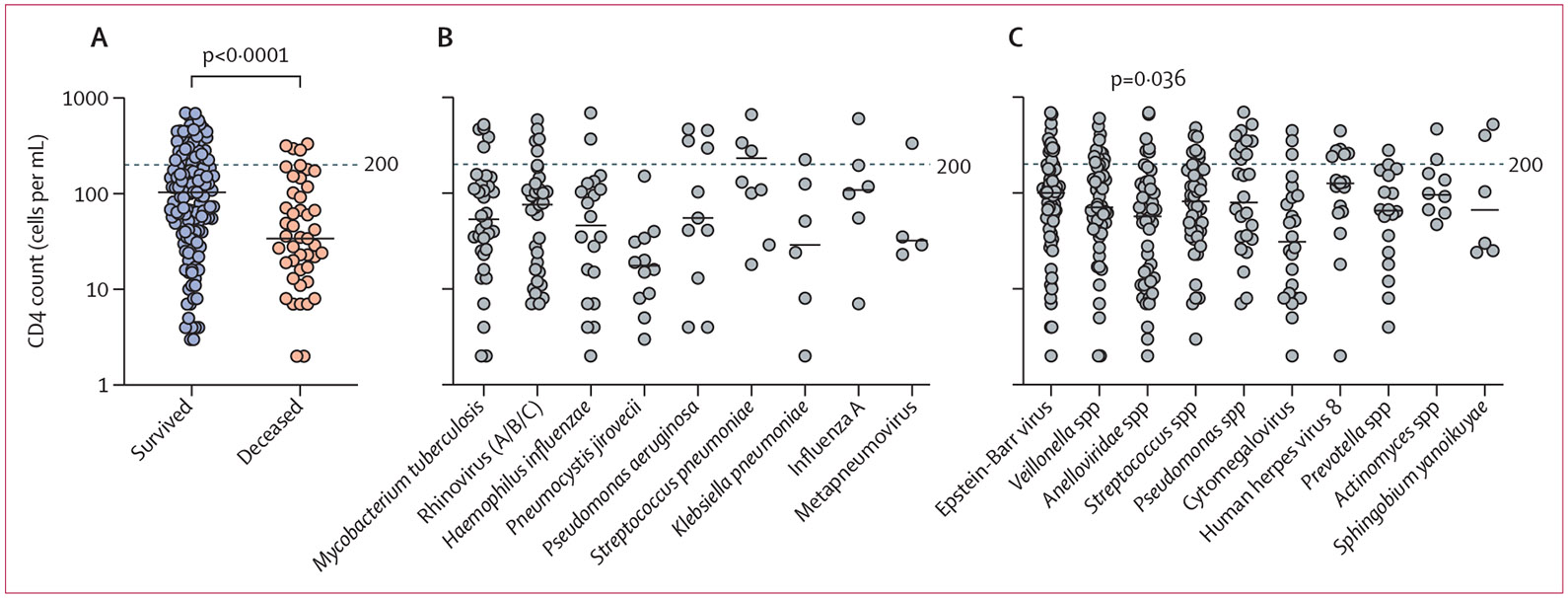
Mortality and pathogen detection by CD4 cell count (A) Baseline CD4 cell counts in patients who were alive at 70 days after enrolment versus in those who had died (Mann-Whitney *U* test, median 103 cells per mL [95% CI 71-136] *vs* 34 [22–62]; p<0·0001). Patients without survival data available were excluded from this analysis. (B) Baseline CD4 cell counts in patients with the most commonly detected established pathogens. (C) Baseline CD4 cell counts in patients with the most common possible pathogens. Significance was defined as a p value of less than 0·05, calculated by Fisher’s exact test. The number of microbes included in each comparison and p values are provided in the appendix (p 25). Each dot represents a single patient and medians are depicted by horizontal bars.

Patients who survived to 70 days after enrolment had significantly higher baseline CD4 cell counts than those who had died by day 70 (figure 3A). Of the established pathogens, *P jirovecii* was detected in 12 [7%] of 166 patients with a CD4 count of less than 200 cells per mL versus none of 51 patients with a CD4 count of 200 cells per mL or greater (Fisher’s exact test p=0·073; figure 3B, appendix p 25). With respect to the possible pneumonia pathogens, Anelloviridae were more commonly identified in patients with a CD4 count of less than 200 cells per mL than in patients with a CD4 count of 200 cells per mL or greater (Fisher’s exact test p=0·036; figure 3C).

**Figure 4:**
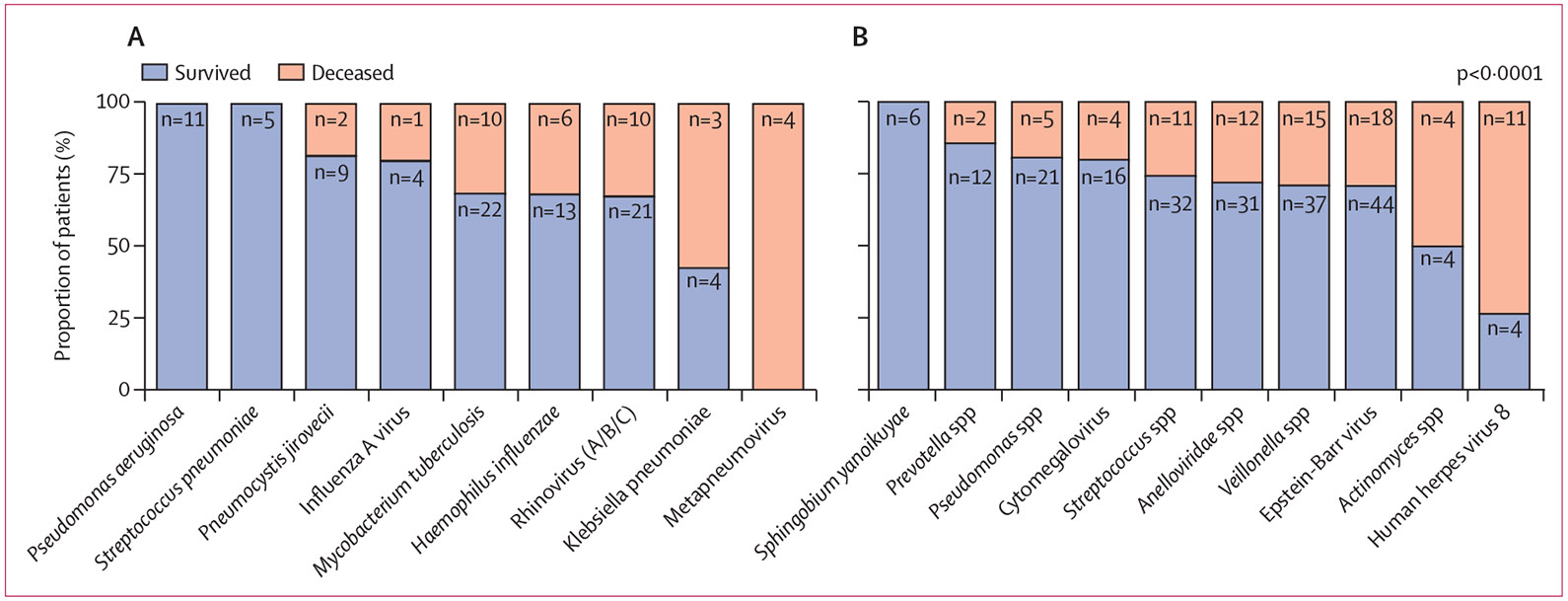
Mortality by pathogen detected (A) Mortality among patients with each of the nine most frequently detected established pathogens. Red indicates death by 70 days after enrolment; blue indicates survival at 70 days. (B) Mortality among patients with each of the ten most frequently detected possible pathogens. Significance was defined as a p value of less than 0·05, calculated by Fisher’s exact test. Patients without mortality data available were excluded from these analyses. The number of microbes included in each comparison and p values are provided in the appendix (p 26).

Human herpes virus 8 was more likely to be found in patients who did not survive to 70 days than in those who were alive at day 70 (Fisher’s exact test p<0·0001; figure 4, appendix p 26).

Interrogating the metatranscriptomic data showed sequences aligning to bacterial antimicrobial resistance genes in 75 (35%) of 217 patients (appendix pp 27-31); tetracycline, aminoglycoside, and β-lactam resistance genes were the most prevalent (appendix p 12). Extended-spectrum β-lactamase genes were identified in 11 (5%) patients.

Patients who were receiving prophylactic trimethoprim–sulfamethoxazole as outpatients before admission to hospital (122 [56%] of 217 patients) were significantly less likely to have *P jirovecii* than those who were not. *P jirovecii* was detected in ten (11%, 95% CI 6–18) of 95 patients not receiving trimethoprim–sulfamethoxazole prophylaxis versus in only two (2%, 0–6) of 122 patients receiving trimethoprim–sulfamethoxazole (Fisher’s exact test p=0·0058; appendix p 32). 14 (11%, 95% CI 7–18) of 122 patients who were receiving trimethoprim–sulfamethoxazole prophylaxis had detectable resistance genes, compared with seven (7%, 4–14) of 95 who were not receiving prophylaxis (Fisher’s exact test p=0·36; appendix p 33).

## Discussion

By utilising culture-independent metatranscriptomic RNA sequencing, we advance understanding of pneumonia causes in the uniquely vulnerable population of people living with HIV, and provide a proof-of-concept for future surveillance studies incorporating this approach. Furthermore, this is one of few studies to date to analyse pneumonia causes using lower respiratory tract sampling in sub-Saharan Africa. Various bacterial, viral, fungal, and mycobacterial respiratory pathogens were identified, including frequent co-infections.

We identified at least one possible pneumonia pathogen in 211 (97%) of 217 patients, and at least one established pneumonia pathogen in 113 (52%) patients. Our rate of pathogen detection compares favourably to a multicentre pneumonia surveillance study in the USA that used more traditional pathogen detection methods and identified a microbiological diagnosis in fewer than half of the study patients,^8^ and a study of community-acquired pneumonia conducted in Malawi between 2013 and 2015 that identified a probable pathogen in 61% of patients with HIV.^19^

Many of the possible pathogens identified in this study, such as the bacterium *T whipplei,* are difficult to identify using conventional methods, but have previously been reported as possible pneumonia pathogens in immunocompromised hosts.^20^ Further work is needed to study such emerging and non-canonical causes of pneumonia. Six patients did not have any pathogens identified, and it is possible that they did not have pneumonia but instead had a condition that can present similarly, such as heart failure exacerbation or a pulmonary embolus.

*M tuberculosis* was the most frequently detected established pathogen in this study, even though patients with clearly positive acid-fast bacilli sputum smears before bronchoalveolar lavage were excluded. *M tuberculosis* was also the most frequently identified pathogen in a 2019 study of Malawian patients with HIV and community-acquired pneumonia.^19^ Surprisingly, the rate of co-infection with other pneumonia pathogens in addition to *M tuberculosis* was 37%, which is higher than in previous reports^19,21^ and is likely to reflect the unbiased assessment of the microbial landscape afforded by metatranscriptomics as compared with conventional culture or PCR assays.^22^ Our findings suggest that even in patients with a diagnosis of *M tuberculosis*, investigation and empirical treatment of other pneumonia pathogens should be considered. Furthermore, in patients with HIV and pneumonia, negative acid-fast bacilli smears might not definitively rule out *M tuberculosis*.

Although the established opportunistic pathogen *P jirovecii* was only found in patients with a CD4 count of less than 200 cells per mL, pathogens responsible for a substantial burden of disease in immunocompetent hosts (eg, *H influenzae,* human metapneumovirus) were also frequently identified in this immunocompromised group of patients with HIV. Not surprisingly, mortality was higher in patients with CD4 counts of less than 200 cells per mL and in patients infected with human herpes virus 8, which is associated with the development of Kaposi sarcoma and primary effusion lymphoma in patients with low CD4 cell counts.

We identified *S mitis* as a potentially clinically important and previously unrecognised pneumonia pathogen in patients with HIV. *S mitis* was the most commonly detected bacterium in terms of either established or possible pathogens, which suggests that it has a putative role as an opportunistic pathogen. Viridians-group streptococci, and *S mitis* in particular, are best known as oral commensal bacteria, but studies have suggested that they can also act as invasive pathogens in immunosuppressed hosts, causing bacteraemia, endocarditis, pneumonia, or other infections in patients with cancer.^23^ *S mitis* has frequently been detected in sputum samples from patients with clinical pneumonia, which further suggests that it has a role as a pathobiont (ie, a microbe that can exist in some contexts as a commensal and in others as a pathogen).^24^

The prevalence of *P aeruginosa* in our cohort was surprising given that it has infrequently been identified as a community-acquired pneumonia pathogen in previous studies;^25^ it merits further attention because antibacterial agents with antipseudomonal activity are rarely used as a first-line pneumonia treatment in Uganda. RNA reads aligning to HIV-1 were detected in 151 (70%) of 217 patients, in line with a previous study that described detection in up to 86% of infected individuals.^26^ Previous work has found that pulmonary infection might enhance HIV replication in the lungs, most notably in cases of *P jirovecii* or *M tuberculosis* pneumonia.^26^

Assessing the human antimicrobial resistome provides an opportunity to understand the burden of potential resistance within populations.^27^ We identified acquired antimicrobial resistance genes in 75 (35%) of 217 patients, and tetracycline and aminoglycoside resistance genes were the most prevalent. We did not find an association between prophylactic treatment with trimethoprim–sulfamethoxazole and detection of trimethoprim and sulfamethoxazole resistance genes in the respiratory microbiome. This study, conducted in 2010, can provide a baseline for future antimicrobial resistance surveillance efforts.^28^

The first clinical laboratory to perform routine RNA sequencing for infectious disease diagnosis was established in 2018 at the University of California, San Francisco (San Francisco, CA, USA).^10^ With the potential to deliver broad-range pathogen surveillance in less than 24 h, clinical metagenomics has directly impacted patient care and permitted the identification of occult, novel, and diagnostically challenging pathogens, which could otherwise be missed by standard testing modalities.^11^ Cost and infrastructure requirements have limited the practicality of deploying metatranscriptomics for routine clinical diagnosis in low-income and middleincome countries. However, given the continued decline in the cost of sequencing and the introduction of openaccess bioinformatics pipelines that reduce the cost and time of data analysis, metatranscriptomics is poised to be applied more broadly for infectious disease epidemiological surveillance.

Although not explored in this study, RNA sequencing can also enable phylogenetic analysis for tracking the emergence and evolution of outbreak pathogens. For instance, during the Ebola virus pandemic, RNA sequencing became crucial for tracking viral transmission patterns in west Africa,^29^ and sequencing has proven instrumental for tracking SARS-CoV-2 variants that have emerged in Africa, Asia, and elsewhere.^30^

Metatranscriptomics provides an opportunity to understand pneumonia causes in cases where traditional techniques have failed, or in settings where the infrastructure needed to perform comprehensive infectious disease epidemiological surveillance is not available. Instead of necessitating laboratory capacity for culture, serology, and antigen and PCR testing, RNA sequencing enables broad-range assessment of multiple pathogens in a single test from a single bronchoalveolar lavage sample. Furthermore, metatranscriptomics has the potential to generate enhanced population-level data that can inform public health policy, ranging from pandemic preparedness to vaccine deployment or adaptation of clinical empirical treatment guidelines.

Our study has some limitations. Enrolment before the COVID-19 pandemic is a limitation, given that both the introduction of SARS-CoV-2 into the population and public health mitigation strategies (eg, face masks) might have lasting effects on pathogen prevalence. Furthermore, the distribution of pneumonia pathogens might differ due to changes in the landscape of HIV infection in Uganda over the past decade. Because of an increase in access to antiretroviral therapy (90% of the population in 2020 *vs* 20% in 2010), HIV incidence in Uganda has decreased from 94 000 to 38 000 new infections per year, while the prevalence has increased from 1·3 million to 1·4 million people infected.^1^

Distinguishing between pathogens and commensals is a major challenge for lower respiratory tract infection diagnostics. RNA sequencing coupled with our rules-based model provides a framework for making this distinction, but further work is needed to more effectively assess the contribution to disease of the identified microbes. High taxonomic abundance does not necessarily equate to pathogenicity, and some bacteria (eg, *S pneumoniae*) can contextually be found as either commensals or pathogens. Furthermore, some pathogens, such as *M tuberculosis*, can generate infection (eg, cavitary disease) without being dominant in the airway. Additionally assessing for microbial virulence factors or profiling the host transcriptome in parallel might overcome some of these challenges and improve the ability to distinguish true infection from colonisation.^9^

Most patients in this cohort were receiving background treatment with antibiotics, which was likely to enrich species that are resistant to first-line antibiotics. However, because detection of nucleic acid by RNA sequencing was the primary diagnostic modality used in this study, our results might be less influenced by concurrent antibiotic administration than microbiological culture would be. Finally, due to the cross-sectional study design and limited outcome data, we were not able to thoroughly assess the impact of RNA sequencing on mortality, although one might expect that targeted treatment of otherwise occult pathogens (eg, *Histoplasma* spp) could improve outcomes.

This study provides a proof-of-concept for culture-independent metatranscriptomics for respiratory pathogen surveillance in a vulnerable population and a region with a high burden of pneumonia.

## Supplementary Material

Supplementary Appendix

## Figures and Tables

**Table:** Baseline characteristics and 70-day mortality

	Patients	Total number of patients assessed
Age, years	36 (10)	217
CD4 count, cells per mL	139 (151)	214
Sex		
Female	131 (60%)	217
Male	86 (40%)	217
Receiving antiretroviral therapy	48 (22%)	217
Receiving *Pneumocystis jirovecii* prophylaxis	122 (56%)	217
Previous *Mycobacterium tuberculosis* diagnosis	16 (7%)	217
Background antibiotic use		
Any antibiotic	183 (84%)	217
Trimethoprim–sulfamethoxazole	138 (63%)	216
Penicillin	84 (39%)	215
Ceftriaxone	80 (37%)	216
Quinolone	16 (7%)	216
Macrolide	40 (18%)	216
In-hospital antibiotic use		
Any antibiotic	185 (85%)	217
Trimethoprim–sulfamethoxazole	147 (68%)	216
Penicillin	60 (28%)	215
Ceftriaxone	89 (41%)	216
Quinolone	11 (5%)	216
Macrolide	29 (13%)	216
70-day mortality	47 (24%)	194

Data are n, n (%), or mean (SD).

## Data Availability

Raw sequencing files containing microbial reads from each of the samples analysed in this study are available under the National Center for Biotechnology Information BioProject accession PRJNA699613. Code for the background correction algorithm is available at https://github.com/czbiohub/idseqr/. Code for the rules-based model and statistical analyses is available at https://github.com/joshbloomstein/rbm-uganda-statistics.

## Research in context

### Evidence before this study

We searched PubMed from database inception to Dec 17, 2021, with no language restrictions for previous pneumonia epidemiological surveillance studies that included people living with HIV in sub-Saharan Africa and incorporated culture-independent metatranscriptomics for pathogen detection. Search terms were “lower respiratory tract infection” AND “pneumonia” AND “epidemiology” AND “HIV” AND “Africa” AND “metagenomic” OR “metatranscriptomic”. Two studies were identified that were unrelated to pneumonia epidemiological surveillance.

### Added value of this study

This proof-of-concept study shows that an unbiased metatranscriptomic approach can be useful in describing the epidemiology of pneumonia causes in the vulnerable demographic of adults living with HIV. This is one of few such studies in sub-Saharan Africa, where there is a disproportionate burden of both HIV and pneumonia. We describe the first use of metatranscriptomics, a culture-independent method for broad-range detection of respiratory pathogens, for epidemiological surveillance of respiratory infections in this demographic. Metatranscriptomics enabled a microbiological diagnosis in 97% of patients, providing a greater understanding on both established and uncommon opportunistic pathogens beyond what has previously been reported. We found that co-infections with viral, bacterial, and fungal or mycobacterial pathogens are common, and occurred in 37% of patients with *Mycobacterium tuberculosis* in this study. We identified *Streptococcus mitis*, a pathobiont not previously appreciated as an opportunistic pathogen in people living with HIV, and found a substantial burden of *Haemophilus influenzae*. Furthermore, we provide an unbiased examination of relationships between CD4 cell count, lower respiratory microbes, and mortality in this population.

### Implications of all the available evidence

Epidemiological surveillance studies are essential for informing empirical treatment approaches in regions with limited clinical microbiologic infrastructure, which disproportionately exist in sub-Saharan African nations, such as Uganda. Here, we explore pneumonia causes in people living with HIV, a demographic that both disproportionately suffers from pneumonia and requires frequent admission to hospital due to severe disease. We found a high prevalence of co-infecting bacterial and viral respiratory pathogens in patients with *M tuberculosis*, emphasising that even with a diagnosis of tuberculosis, investigation and empirical treatment of other pneumonia pathogens should be considered, and vice versa. We identified respiratory viruses and bacterial species not previously associated with low CD4 cell count, expanding our existing understanding of opportunistic pneumonia pathogens.

## References

[1] UN. UNAIDS country factsheet. 2020. https://www.unaids.org/en/resources/documents/2020/unaids-data (accessed July 5, 2021).

[2] Cohen C, Walaza S, Moyes J, Epidemiology of severe acute respiratory illness (SARI) among adults and children aged ≥5 years in a high HIV-prevalence setting, 2009–2012. PLoS One 2015; 10: e0117716. 25706880 10.1371/journal.pone.0117716 PMC4337909

[3] Marshall CS, Curtis AJ, Spelman T, Impact of HIV-associated conditions on mortality in people commencing anti-retroviral therapy in resource limited settings. PLoS One 2013; 8: e68445. 23935870 10.1371/journal.pone.0068445 PMC3720807

[4] Kyeyune R, den Boon S, Cattamanchi A, Causes of early mortality in HIV-infected TB suspects in an east African referral hospital. J Acquir Immune Defic Syndr 2010; 55: 446–50. 21105258 10.1097/qai.0b013e3181eb611a PMC3249444

[5] Prina E, Ranzani OT, Torres A. Community-acquired pneumonia. Lancet 2015; 386: 1097–108. 26277247 10.1016/S0140-6736(15)60733-4 PMC7173092

[6] Theodoratou E, McAllister DA, Reed C, Global, regional, and national estimates of pneumonia burden in HIV-infected children in 2010: a meta-analysis and modelling study. Lancet Infect Dis 2014; 14: 1250–58. 25455992 10.1016/S1473-3099(14)70990-9 PMC4242006

[7] Nantanda R, Hildenwall H, Peterson S, Kaddu-Mulindwa D, Kalyesubula I, Tumwine JK. Bacterial aetiology and outcome in children with severe pneumonia in Uganda. Ann Trop Paediatr 2008; 28: 253–60. 19021940 10.1179/146532808X375404

[8] Jain S, Self WH, Wunderink RG, Community-acquired pneumonia requiring hospitalization among U.S. adults. N Engl J Med 2015; 373: 415–27. 26172429 10.1056/NEJMoa1500245 PMC4728150

[9] Langelier C, Kalantar KL, Moazed F, Integrating host response and unbiased microbe detection for lower respiratory tract infection diagnosis in critically ill adults. Proc Natl Acad Sci USA 2018; 115: e12353–62. 30482864 10.1073/pnas.1809700115 PMC6310811

[10] Wilson MR, Sample HA, Zorn KC, Clinical metagenomic sequencing for diagnosis of meningitis and encephalitis. N Engl J Med 2019; 380: 2327–40. 31189036 10.1056/NEJMoa1803396 PMC6764751

[11] Greninger AL, Naccache SN, Federman S, Rapid metagenomic identification of viral pathogens in clinical samples by real-time nanopore sequencing analysis. Genome Med 2015; 7: 99. 26416663 10.1186/s13073-015-0220-9 PMC4587849

[12] Saha S, Ramesh A, Kalantar K, Unbiased metagenomic sequencing for pediatric meningitis in Bangladesh reveals neuroinvasive chikungunya virus outbreak and other unrealized pathogens. MBio 2019; 10: e02877–19. 31848287 10.1128/mBio.02877-19 PMC6918088

[13] Shenoy MK, Iwai S, Lin DL, Immune response and mortality risk relate to distinct lung microbiomes in patients with HIV and pneumonia. Am J Respir Crit Care Med 2017; 195: 104–14. 27447987 10.1164/rccm.201603-0523OC PMC5214918

[14] Iwai S, Huang D, Fong S, The lung microbiome of Ugandan HIV-infected pneumonia patients is compositionally and functionally distinct from that of San Franciscan patients. PLoS One 2014; 9: e95726. 24752365 10.1371/journal.pone.0095726 PMC3994144

[15] Kalantar KL, Carvalho T, de Bourcy CFA, IDseq-An open source cloud-based pipeline and analysis service for metagenomic pathogen detection and monitoring. Gigascience 2020; 9: giaa111. 33057676 10.1093/gigascience/giaa111 PMC7566497

[16] Magill SS, O’Leary E, Ray SM, Antimicrobial use in US hospitals: comparison of results from emerging infections program prevalence surveys, 2015 and 2011. Clin Infect Dis 2021; 72: 1784–92. 32519751 10.1093/cid/ciaa373 PMC7976440

[17] Mandell LA, Wunderink RG, Anzueto A, Infectious Diseases Society of America/American Thoracic Society consensus guidelines on the management of community-acquired pneumonia in adults. Clin Infect Dis 2007; 44 (suppl 2): S27–72. 17278083 10.1086/511159 PMC7107997

[18] Fishman JA. Infection in organ transplantation. Am J Transplant 2017; 17: 856–79. 28117944 10.1111/ajt.14208

[19] Aston SJ, Ho A, Jary H, Etiology and risk factors for mortality in an adult community-acquired pneumonia cohort in Malawi. Am J Respir Crit Care Med 2019; 200: 359–69. 30625278 10.1164/rccm.201807-1333OC PMC6680311

[20] Lozupone C, Cota-Gomez A, Palmer BE, Widespread colonization of the lung by *Tropheryma whipplei* in HIV infection. Am J Respir Crit Care Med 2013; 187: 1110–17. 23392441 10.1164/rccm.201211-2145OC PMC3734615

[21] Vray M, Germani Y, Chan S, Clinical features and etiology of pneumonia in acid-fast bacillus sputum smear-negative HIV-infected patients hospitalized in Asia and Africa. AIDS 2008; 22: 1323–32. 18580612 10.1097/QAD.0b013e3282fdf8bf

[22] Schleicher GK, Feldman C. Dual infection with *Streptococcus pneumoniae* and *Mycobacterium tuberculosis* in HIV-seropositive patients with community acquired pneumonia. Int J Tuberc Lung Dis 2003; 7: 1207–08. 14677897

[23] Shelburne SA, Sahasrabhojane P, Saldana M, *Streptococcus mitis* strains causing severe clinical disease in cancer patients. Emerg Infect Dis 2014; 20: 762–71. 24750901 10.3201/eid2005.130953 PMC4012796

[24] Musher DM, Jesudasen SS, Barwatt JW, Cohen DN, Moss BJ, Rodriguez-Barradas MC. Normal respiratory flora as a cause of community-acquired pneumonia. Open Forum Infect Dis 2020; 7: ofaa307. 32968689 10.1093/ofid/ofaa307 PMC7491709

[25] Gadsby NJ, Russell CD, McHugh MP, Comprehensive molecular testing for respiratory pathogens in community-acquired pneumonia. Clin Infect Dis 2016; 62: 817–23. 26747825 10.1093/cid/civ1214 PMC4787606

[26] Koziel H, Kim S, Reardon C, Enhanced in vivo human immunodeficiency virus-1 replication in the lungs of human immunodeficiency virus-infected persons with *Pneumocystis carinii* pneumonia. Am J Respir Crit Care Med 1999; 160: 2048–55. 10588627 10.1164/ajrccm.160.6.9902099

[27] Kim DW, Cha CJ. Antibiotic resistome from the One-Health perspective: understanding and controlling antimicrobial resistance transmission. Exp Mol Med 2021; 53: 301–09. 33642573 10.1038/s12276-021-00569-z PMC8080597

[28] WHO. Antimicrobial resistance: global report on surveillance. 2014. https://apps.who.int/iris/handle/10665/112642 (accessed July 30, 2021).

[29] Quick J, Loman NJ, Duraffour S, Real-time, portable genome sequencing for Ebola surveillance. Nature 2016; 530: 228–32. 26840485 10.1038/nature16996 PMC4817224

[30] Lamptey J, Oyelami FO, Owusu M, Genomic and epidemiological characteristics of SARS-CoV-2 in Africa. PLoS Negl Trop Dis 2021; 15: e0009335. 33901167 10.1371/journal.pntd.0009335 PMC8101992

