# Supplementary material for: Pneumonia surveillance with culture-independent metatranscriptomics in HIV-positive adults in Uganda: a cross-sectional study
Source: Lancet Microbe. Author manuscript; Available in PMC 2025 Jun 25. (PMC12193104; doi:10.1016/S2666-5247(21)00357-8)
Supplement: Supplementary Appendix [file NIHMS2088302-supplement-Supplementary_Appendix.pdf]

THE LANCET
Microbe

Supplementary appendix

This appendix formed part of the original submission and has been peer reviewed. We post it as supplied by the authors.

Supplement to: Spottiswoode N, Bloomstein JD, Caldera S, et al. Pneumonia surveillance with culture-independent metatranscriptomics in HIV-positive adults in Uganda: a cross-sectional study. *Lancet Microbe* 2022; published online March 25. https://doi.org/10.1016/S2666-5247(21)00357-II.

Supplementary Appendix

Pneumonia Surveillance in HIV-Infected Adults in Uganda Through the Lens of Culture-Independent Metatranscriptomics: A Cross Sectional Study

Table of Contents

Supplementary Methods

Supplementary Results

Supplementary Figures

Supplementary Figure 1. Distinguishing LRTI pathogens from commensal respiratory microbiota using the rules-based model (RBM).
Supplementary Figure 2. Co-infection prevalence among established and possible pathogens
Supplementary Figure 3. Relative distribution of lower respiratory tract antimicrobial resistance (AMR) genes by class

Supplementary Tables

Supplementary Table 1. List of established pathogens derived from pneumonia epidemiological surveillance studies and clinical guidelines[1]
Supplementary Table 2: Overview of pathogens identified by RNA-seq as well as MTB and PJP clinical diagnostics.
Supplementary Table 3. *Mycobacterium tuberculosis* diagnostics.
Supplementary Table 4. Established pathogens detected in patients.
Supplementary Table 5. Summary of microbes co-detected with *Mycobacterium tuberculosis.*
Supplementary Table 6. Detection frequency of microbes identified as possible pneumonia pathogens.
Supplementary Table 7. Comparison between RNA-seq and viral multiplex PCR
Supplementary Table 8. Comparison between RNA-seq and 16s rRNA gene sequencing for bacterial taxa detected by the rules-based model.
Supplementary Table 9. Data and statistical calculations for CD4 count and pathogen detection associated with Figure 3.
Supplementary Table 10. Data and statistical calculations for mortality and pathogen detection associated with Figure 4.
Supplementary Table 11. Lower respiratory tract antimicrobial resistance genes identified in each patient.
Supplementary Table 12. Incidence of *Pneumocystis jirovecii* and receipt of TMP-SMX prophylaxis.
Supplementary Table 13. Trimethoprim-sulfamethoxazole (TMP-SMX) antimicrobial resistance (AMR) genes detected and receipt of TMP-SMX prophylaxis (ppx). A) 2 x 2 matrix of TMP-SMX ppx versus AMR genes detected. B) TMP and SMX AMR genes detected.
Supplementary Table 14. Patient enrollment details.

Supplementary References

Supplementary Methods

Analysis approach and study rationale

Prior lung microbiome studies of this cohort that incorporated 16S rRNA gene sequencing[1,2] suggested unique pneumonia microbiology in Ugandan HIV patients. Such studies were limited, however, by the inability to detect RNA viruses or fungal pathogens, and the inability to detect transcriptionally-active microbiota. Further, robust bioinformatics approaches for identifying bacterial, fungal and viral respiratory pathogens from metagenomic data[3,4] had not been developed at the time. Given the dearth of pneumonia epidemiological surveillance studies in both this vulnerable demographic and geographic region, and the potential for metagenomic RNA sequencing to broaden our understanding of disease epidemiology, the present study was carried out. While our primary objective was to identify putative microbial etiologies of pneumonia within the cohort, we recognized that these analyses could also provide a foundation for future studies of the respiratory virome and transcriptome.

Patient enrollment

Inclusion criteria were age ≥ 18, diagnosis of HIV, clinically diagnosed pneumonia based on cough and an abnormal chest radiograph, and available bronchoalveolar lavage (BAL) fluid RNA for analysis. Exclusion criteria were inability for patient or surrogate to provide informed consent, contraindication, or inability to perform bronchoscopy, existing clinical diagnosis of TB or positive AFB smears, and age < 18 years. BAL fluid was available for nucleic acid extraction from 217 of 642 patients, and was also utilized in two previously published lung microbiome studies[1,2]. Patient enrollment details are summarized in (**Supplementary Table 14**). Bronchoscopies were performed within 72 hours of enrollment. The endpoint of the study was survival at 70 days post-bronchoscopy.

Sample collection

Fiberoptic bronchoscopy with BAL was performed by an experienced chest physician a median of three days after hospital admission (interquartile range, 1–4 days). BAL was performed in the sub-segment that the bronchoscopist deemed to have the most opacities on chest radiograph, or preferentially in the right middle lobe if the radiograph revealed diffuse disease. Prior to nucleic acid extraction, BAL was sent for AFB smear and mycobacterial culture on solid Lowenstein-Jensen media as well as microscopic examination for *Pneumocystis jirovecii*. Patients were considered MTB positive if they had >50 colony forming units (CFU) in sputum or BAL cultures, a positive GeneXpert, and/or were positive for MTB by RNA-seq. Fifteen mL of BAL fluid kept on ice was mixed with 30 mL of RNAlater solution (Life Technologies, Carlsbad, CA, USA) and placed at 4°C for 16 hours, prior to storage at −80°C until shipping from Uganda to San Francisco on dry ice in batches approximately every six months. Upon receipt, samples were stored at −80°C until RNA extraction.

We employed BAL as a diagnostic technique because it overcomes challenges with oropharyngeal microbiota contamination that might be expected in sputum or saliva specimens[5]. BAL has historically been considered the gold-standard for diagnosis of PJP[6] and other types of pneumonia, and the rules-based model employed in this study was developed and validated on lower respiratory tract specimens, although future work will aim to test applicability of the rules-based model (RBM) on RNA-seq data from sputum and upper respiratory samples.

Nucleic acid extraction and RNA sequencing

Nucleic acid extraction from whole BAL was carried out as described in previous studies[1,2,7]. More specifically, thawed BAL samples were centrifuged at 5,000x g, the supernatant was removed, pellets were resuspended in sterile PBS, and centrifuged again at high speed prior to

extraction. Cells were lysed by bead-beating using a FastPrep system (MP Biomedicals, Irvine, CA, USA) for 30 seconds at 5.5 m/s. RNA was then extracted from cell pellets using the Allprep DNA/RNA kit (Qiagen, Germantown, MD, USA) according to the manufacturer's instructions. Residual DNA was removed with on-column DNAse (Qiagen, Germantown, MD, USA) according to manufacturer's instructions, and purified RNA was eluted in nuclease-free water.

RNA quality was assessed as previously described [1,2,7] and then carried forward for library construction using the NEBNext Ultra II Library Prep Kit (New England Biolabs, Ipswich, MA, USA), which incorporates RNA fragmentation, reverse transcription to generate complementary DNA (cDNA), adapter ligation and PCR amplification. The final RNA-Seq libraries were pooled in equimolar amounts and size-selected to retain 300-500 base-pair fragments. Appropriate size and concentration of the finished libraries was confirmed by Bioanalyzer (Agilent, Santa Clara, CA, USA) before undergoing 150 nucleotide paired-end Illumina sequencing on a NovaSeq 6000 (Illumina, San Diego, CA, USA). All 217 specimens yielded quality sequencing data, with a median read count of 8.5×10^7 (95% confidence interval (CI) 8.4×10^7 to 8.6×10^7).

Detection of respiratory microbes using IDseq

Detection of microbes leveraged the open-source IDseq pipeline[4] which incorporates subtractive alignment of the human genome (NCBI GRC h38) using STAR[8], quality filtering using PRICESeqfilter[9], and removal of additional non-fungal eukaryotes, cloning vectors and phiX phage using Bowtie2[10]. The identities of the remaining microbial reads are determined by querying the NCBI nucleotide (NT) and non-redundant protein (NR) databases using GSNAP-L and RAPSEARCH2, respectively.

IDseq classifies all sequencing reads at the species level[4]. In cases where a read maps equally well to multiple different species due to sequence homology, the read is randomly assigned. In

most cases of pneumonia, there is a dominant microbial species, but frequently (due to sequence homology within bacterial genera) a small number of reads are assigned to other species within the genus. To reduce the impact of nonspecific reads being mis-attributed to other species and to focus on the dominant species within each genus, the RBM only evaluates the dominant representative from each species. The chance of a species-level misidentification is very low, but could theoretically happen if two similar species within the same dominant genus (e.g *S. pneumoniae and S.* mitis) were present at comparable frequency.

Rules-based model (RBM) for identifying pathogens amidst a background of commensal respiratory microbiota.

The RBM takes advantage of the collapse in airway microbiome diversity that occurs in the context of pathogen dominance during LRTI (**Supplementary Figure 1**). Before RBM implementation, background correction was carried out (described below) to retain taxa that were statistically significantly different in abundance versus negative water controls. The RBM first identified all human viruses detected at >0.1 rpM. Then, the bacterial and fungal taxa were aggregated to the genus level and sorted in descending order by abundance measured in RNA-seq reads per million (rpM), independently for each sample.

As performed in the initial validation study[3], the greatest gap in abundance between sequentially ranked microbes in each sample was identified (**Supplementary Figure 1**). All genera with an abundance greater than this largest gap threshold were then evaluated at the species level, by identifying the most abundant species within each genus. If the species was present within a previously developed reference index of established respiratory pathogens derived from landmark epidemiologic surveillance studies and clinical guidelines[3,11–15] (**Supplementary Table 1**), it was selected as an 'established' pathogen by the RBM. Viral, bacterial and fungal taxa identified by the RBM but not included on the reference list of established respiratory pathogens

were considered possible respiratory pathogens. Example cases are highlighted in (**Supplementary Figure 1**). Prior work has demonstrated that RNA-seq in combination with the RBM enables accurate pathogen identification even in patients who have received antibiotics prior to sample collection[3,16,17], since nucleic acid, including bacterial ribosomal RNA, may persist even after bacterial replication ceases.

Identification and mitigation of environmental contaminants

To minimize inaccurate taxonomic assignments due to environmental contaminants, we 1) processed a negative water control with the sequencing run, and 2) spiked External RNA Controls Consortium (ERCC) positive control standards into each sample[18,19]. ERCC RNA standards, a mixture of transcripts derived from US National Institute of Standards and Technology-certified DNA plasmids, were spiked into all samples at a pre-determined mass of 25pg to permit calculation of microbial RNA mass in each sample according to the following equation: (ERCC mass input/microbe mass) = (ERCC sequencing reads/microbe sequencing reads). Microbial mass calculation is utilized in our background correction model that assesses the statistical significance of microbial sequencing alignments in the patient samples compared to those in negative control samples sequenced in parallel.

NT_r reads aligning to unidentifiable or ubiquitous contaminant taxa[20] found in metagenomic library preparation reagents were filtered from all samples (e.g. *Bradyrhizobium* species, *Ralstonia* species). Bacteriophages plants and insects were excluded. For each sample, a normalized background subtraction was then performed at the nucleotide level. Background subtraction was performed using a recently validated negative binomial model[2]. For each sample NT_r reads were summed at the genus level. Within each genus the species with the most NT_r reads was designated as the microbe present.

Detection of antimicrobial resistance genes

To identify antimicrobial resistance genes present in the metagenomic dataset, the SRST2 computational program and Argannot2 database were employed[21], with results listed in (**Supplementary Table 11**). AMR genes derived from background environmental contamination present in negative control samples were excluded. AMR genes known to confer trimethoprim or sulfa resistance (e.g. *DfrA, Sul1*) were specifically evaluated to determine whether a significance difference in the prevalence of these resistance genes existed between patients who did or did not receive prophylactic treatment with trimethoprim/sulfamethoxazole (**Supplementary Table 13**).

Confirmatory respiratory virus testing by multiplex PCR

We employed the Luminex XTAG PCR platform, which is clinically validated for detection of influenza, RSV, adenovirus, parainfluenza virus 1-4 and rhinovirus, to confirm RNA-seq findings. We tested cDNA derived from 20 representative specimens that had a respiratory virus detected by RNA-seq. Data are tabulated in (**Supplementary Table 7**).

Comparison with 16S rRNA gene sequencing data

Samples with established or possible bacterial pathogens called by the RBM that also had 16S rRNA gene sequencing data available from prior microbiome studies[1,2] were evaluated (n = 142 samples, n = 198 taxa). The rank based on taxon relative abundance in the 16S data for each bacterial taxon detected by RNA-seq using the RBM was identified and tabulated in (**Supplementary Table 8**).

Differential Analysis by CD4 Count

For each sample, NT_r reads of the top 10 most abundant microbes with NT_rpm > 0·1 were assessed. Patients were stratified by CD4 count and differential abundance at the genus level was compared in those patients with CD4 <200 cells/μL versus patients with CD4 ≥200 cells/μL. For each genus, the proportion of patients with the microbe present was calculated. The difference in proportions between groups was calculated. For each taxon, Fisher's exact test was used to test for statistical significance of the difference in abundance between groups. Benjamini-Hochberg method was used to calculate adjusted p values.

Supplementary Results

Confirmation of RNA-seq results

We pursued several approaches to independently assess RNA-seq findings. First, we performed multiplex reverse-transcriptase polymerase chain reaction (RT-PCR) for 12 respiratory viruses on a representative subset (n = 20) of samples with viruses detected by mNGS. We found 100% concordance between RNA-seq results and RT-PCR. (**Supplementary Table 7**). Because Giemsa staining was performed for *Pneumocystis jirovecii* (PJP) on all specimens, we also assessed the performance of RNA-seq + RBM for detecting this opportunistic fungal pathogen. RNA-seq + mNGS detected eight (100%) of PJP cases identified by Giemsa stain, and four additional cases that were missed by Giemsa staining. We additionally compared the bacterial taxa identified by RNA-seq + RBM against 16S rRNA gene DNA sequencing data from prior microbiome studies of this cohort[1,2]. Of 142 subjects with one or more bacterial taxa identified by RNA-seq, we found that in 124 (87·3%), an RNA-seq + RBM-identified microbe was within the top 10 most abundant microbes detected by 16S, and 105 (73·9%) within the top three. With respect to MTB, RNA-seq combined with the RBM only detected three (8·5%) cases (**Supplementary Table 8**).

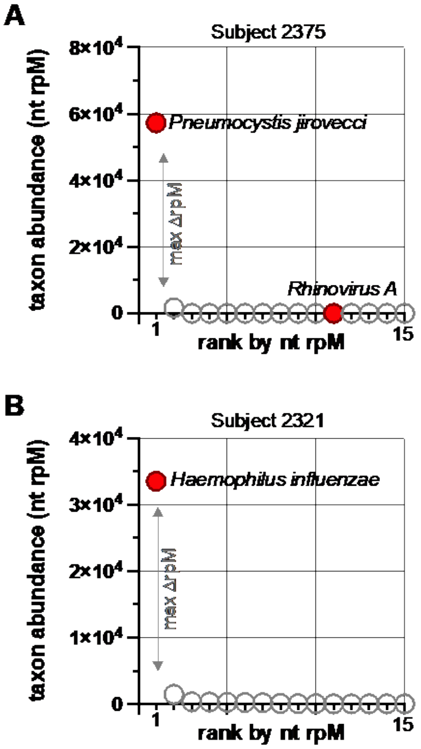

Supplementary Figure 1. Distinguishing LRTI pathogens from commensal respiratory microbiota using the rules-based model (RBM). Microbial relative abundance in BAL RNA-seq reads per million reads sequenced (rpM) by RNAseq for two representative cases. A) A patient with definite LRTI from whom *P. jirovecii* and Rhinovirus A were identified by the RBM. B) A patient from whom *H. influenzae* was detected by the RBM. For all cases, the largest abundance differential between ranked microbes (max Δ rpM) was used as a threshold to identify high-scoring taxa. Red indicates taxa represented in the reference list of established LRTI pathogens (**Supplementary Table 1**), called as 'established pathogens' by the RBM.

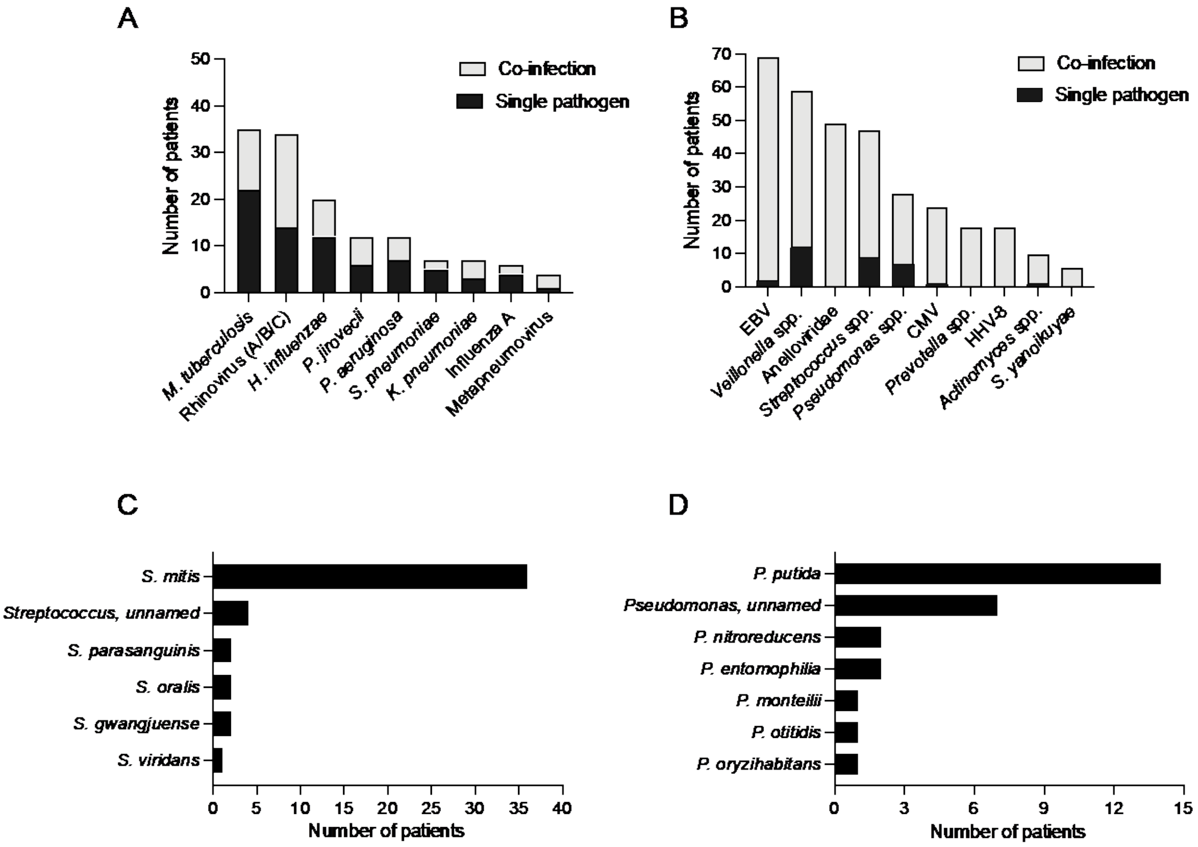

Supplementary Figure 2. Co-infections are common among established and possible pathogens. (A) Most frequent established pathogens identified; light grey indicates co-infection with another established pathogen; black indicates only one established pathogen detected. (B) Most frequent possible pathogens identified; light grey indicates co-infection with another established or possible pathogen, black indicates it is the only pathogen detected. (C) *Streptococcal* pathogens detected, excluding established pathogen *S. pneumonia*. (D) *Pseudomonal* pathogens detected, excluding *P. aeruginosa.*

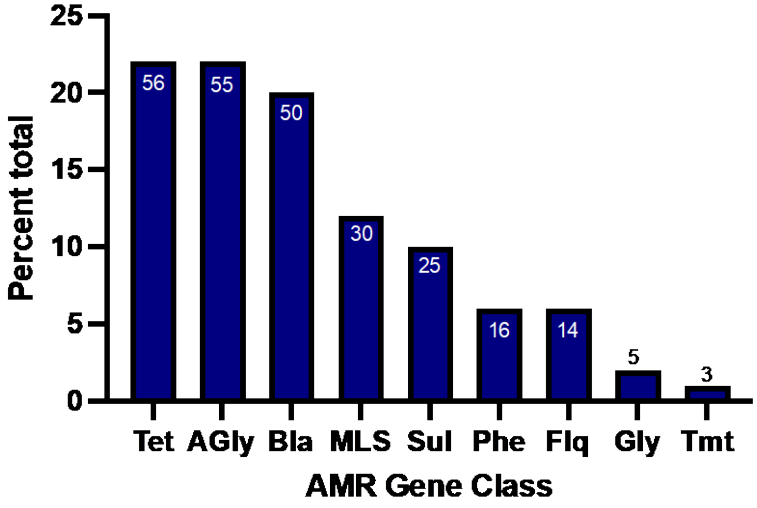

Supplementary Figure 3. Relative distribution of lower respiratory tract antimicrobial resistance (AMR) genes by class. Total number of acquired AMR genes detected = 254. Tet = tetacycline, AGly = aminoglycoside, Bla = beta-lactamase, MLS = macrolide, lincosomide, streptogramin, Sul = sulfa, Phe = phenicol, Flq = fluoroquinolone, Gly = glycopeptide, Tmt = trimethoprim.

Supplementary Table 1. Reference index of established respiratory pathogens derived from epidemiological surveillance studies and clinical guidelines[3,11–15].

**Reference Index of Established Pneumonia Pathogens**
**Bacteria and Fungi**
Acinetobacter baumannii
Bacteroides fragilis
Bordatella pertussis
Burkholderia cepacia
Burkholderia pseudomallei
Chlamydophila pneumoniae
Chlamydophila psittaci
Citrobacter fruendii
Citrobacter koseri
Coxiella burnetii
Enterobacter aerogenes
Enterobacter cloacae
Escherichia coli
Francisella tularemia
Fusobacterium necrophorum
Fusobacterium nucleatum
Haemophilus influenzae
Klebsiella oxytoca
Klebsiella pneumoniae
Legionella pneumophila
Moraxella catarrhalis
Morganella morganii
Mycobacterium tuberculosis
Mycoplasma pneumoniae
Nocardia spp.
Pasturella multocida
Proteus mirabilis
Pseudomonas aeruginosa
Serratia marcescens
Stenotrophomonas maltophilia
Streptococcus pneumoniae
Streptococcus pyogenes
Staphylococcus aureus

**Viruses**
Human adenovirus
Human coronavirus
Human metapneumovirus
Human parainfluenza virus
Human rhinovirus
Influenza virus
Respiratory syncytial virus
**Fungi**
Aspergillus flavus
Aspergillus fumigatus
Aspergillus niger
Aspergillus terreus
Cryptococcus gatii
Cryptococcus neoformans
Coccidioides imitis
Coccidioides posadisii
Histoplasma capsulatum
Blastomyces dermatitidis
Pneumocystis jirovecii
Rhizopus, Mucor and Rhizomucor spp.

Supplementary Table 2. Overview of pathogens identified by RNA-seq as well as MTB and PJP clinical diagnostics.

	Patients n (%)
**Patients enrolled**	217
Sequencing data available	217 (100%)
Established pathogen identified	113 (52%)
Possible pathogen identified	98 (45%)
No pathogen identified	6 (3%)
**Patients with established pathogens (n=113)**	
Bacterial pathogens	48 (43%)
Viral pathogens	49 (43%)
Mycobacterial pathogens	35 (31%)
Fungal pathogens	13 (12%)
Only established bacterial pathogens detected	30 (27%)
Only established viral pathogens detected	26 (23%)
Only established mycobacterial pathogens detected	22 (20%)
Only established fungal pathogens detected	7 (6%)
More than one pathogen class detected	28 (25%)

Supplementary Table 3. ***Mycobacterium tuberculosis*** **diagnostics.**

Patient ID	Sputum culture positive	BAL culture positive	Gene Xpert	RNA-Seq + RBM
**1153**	1	0	1	0
**1186**	1	0	1	0
**1290**	NA	1	NA	0
**1292**	1	0	0	0
**1325**	0	1	NA	0
**1328**	0	1	NA	0
**1390**	0	0	1	0
**1397**	1	0	1	0
**1416**	1	0	0	0
**1424**	1	0	1	0
**1428**	NA	NA	NA	1
**1436**	1	0	0	0
**1442**	1	0	1	0
**1444**	NA	0	1	0
**1477**	0	1	1	0
**1488**	1	0	NA	0
**1499**	1	0	NA	0
**1541**	0	1	0	0
**1618**	1	0	0	0
**1674**	NA	1	NA	1
**1689**	1	0	NA	0
**1720**	NA	1	NA	0
**1728**	1	0	NA	0
**1774**	1	0	NA	0
**1776**	1	0	NA	0
**1784**	1	0	NA	0
**1793**	1	0	NA	0
**1795**	1	0	NA	0
**1813**	1	1	NA	0
**1867**	1	1	NA	0
**1905**	NA	0	NA	1
**2032**	0	0	1	0
**2044**	1	0	NA	0
**2164**	0	0	1	0
**2208**	1	0	0	0

Each patient shown has at least one positive test. 1 indicates ≥1 positive test in the column category; "0" indicates all tests were negative; and "NA" indicates that the indicated tests were not performed or results were not available. RNA-seq + RBM = Metatranscriptomic next-generation sequencing combined with the rules based model for pathogen detection. MTB = *Mycobacterium tuberculosis*. rpM = reads per million reads sequence. Culture positive defined as >50 CFU.

222 **Supplementary Table 4. Established pathogens detected in patients.**

Established pathogens	Sole established pathogen detected (*n, %*)	Present as co-infection (*n, %*)	Total number of patients with pathogen (*n, %*)
**Bacterial pathogens**			
Escherichia coli	1 (1%)	1 (1%)	2 (1%)
Haemophilus influenzae	12 (6%)	8 (4%)	20 (9%)
Klebsiella pneumoniae	3 (1%)	4 (2%)	7 (3%)
Mycoplasma pneumoniae	0 (0%)	1 (1%)	1 (1%)
Neisseria meningitidis	0 (0%)	1 (1%)	1 (1%)
Pseudomonas aeruginosa	7 (3%)	5 (2%)	12 (6%)
Stenotrophomonas maltophilia	1 (1%)	0 (0%)	1 (6%)
Streptococcus pneumoniae	5 (2%)	2 (1%)	7 (3%)
**Viral pathogens**			
Coronavirus HKU1	1 (1%)	0 (0%)	1 (1%)
Coronavirus 229E	1 (1%)	0 (0%)	1 (1%)
Influenza A	4 (2%)	2 (1%)	6 (3%)
Influenza B	1 (1%)	0 (0%)	1 (1%)
Metapneumovirus	1 (1%)	3 (1%)	4 (2%)
Parainfluenza virus 2*	1 (1%)	0 (0%)	1 (1%)
Parainfluenza virus 3*	1 (1%)	0 (0%)	1 (1%)
Parainfluenza virus 4*	1 (1%)	0 (0%)	1 (1%)
Respiratory syncytial virus	0 (0%)	1 (1%)	1 (1%)
Rhinovirus A*	6 (3%)	10 (5%)	16 (7%)
Rhinovirus B*	5 (2%)	6 (3%)	11 (5%)
Rhinovirus C*	3 (1%)	6 (3%)	9 (4%)
**Mycobacterial pathogens**			
Mycobacterium tuberculosis	22 (10%)	13 (6%)	35 (16%)
**Fungal pathogens**			
Pneumocystis jirovecii	6 (3%)	6 (3%)	12 (6%)
Histoplasma capsulatum	1 (1%)	0 (0%)	1 (1%)

223 ***Combined at the genus level for further analyses**

Supplementary Table 5. Summary of microbes co-detected with *Mycobacterium tuberculosis.*

	**N patients (percentage)**
**Patients with *M. tuberculosis detected***	35
M. tuberculosis only pathogen detected	22 (63%)
**Co-infection with established pathogen**	13 (38%)
Rhinovirus (A/B/C)	6 (17%)
Pseudomonas aeruginosa	4 (11%)
Haemophilus influenzae	3 (9%)
Klebsiella pneumoniae	1 (3%)
Neisseria meningitidis	1 (3%)
Metapneumovirus	2 (6%)
Pneumocystis jirovecii	2 (6%)
**3+ established pathogens**	4 (14%)

Supplementary Table 6. Detection frequency of microbes identified as possible pneumonia pathogens.

Possible pathogen	Sole pathogen detected (*n, %*)	Present as co-infection *(n, %)*	Patients with pathogen (*n, %*)
Achromobacter spp.	0 (0%)	2 (1%)	2 (1%)
Acinetobacter schindleri	1 (1%)	0 (0%)	1 (1%)
Actinomyces spp.	1 (1%)	9 (4%)	10 (5%)
Aichivirus	0 (0%)	3 (1%)	3 (1%)
Alloprevatella spp,	0 (0%)	2 (1%)	2 (1%)
Anaerococcus prevotii	0 (0%)	1 (1%)	1 (1%)
Anelloviridae family	0 (0%)	49 (23%)	49 (23%)
Burkholderia ambifara	0 (0%)	1 (1%)	1 (1%)
Campylobacter concisus	0 (0%)	2 (1%)	2 (1%)
Capnocytophaga gingivalis	0 (0%)	2 (1%)	2 (1%)
Corynebacterium kroppenstedtii	0 (0%)	1 (1%)	1 (1%)
Cryptosporidium parvum	1 (1%)	1 (1%)	2 (1%)
Enterobacter kobei	0 (0%)	2 (1%)	2 (1%)
Enterococcus faecalis	0 (0%)	1 (1%)	1 (1%)
Enterovirus B	0 (0%)	1 (1%)	1 (1%)
Finegoldia magna	0 (0%)	1 (1%)	1 (1%)
Fusobacterium spp.	0 (0%)	2 (1%)	2 (1%)
Haemophilus spp.*	0 (0%)	2 (1%)	2 (1%)
Hathewaya histolytica	0 (0%)	1 (1%)	1 (1%)
Human alphaherpesvirus 1 (HSV-1)	0 (0%)	5 (2%)	5 (2%)
Human betaherpesvirus 5 (CMV)	1 (1%)	23 (11%)	24 (11%)
Human betaherpesvirus 6 (HHV-6)	0 (0%)	2 (1%)	2 (1%)
Human gammaherpesvirus 4 (EBV)	2 (1%)	67 (31%)	69 (32%)
Human gammaherpesvirus 8 (HHV-8)	0 (0%)	18 (8%)	18 (8%)
Hepatitis B virus	0 (0%)	3 (1%)	3 (1%)
Human immunodeficiency virus 1	0 (0%)	151 (70%)	151 (70%)
Lactobacillus spp.	0 (0%)	5 (2%)	5 (2%)
Leptotrichia spp.	0 (0%)	3 (1%)	3 (1%)
Mycoplasma spp.*	0 (0%)	2 (1%)	2 (1%)
Neisseria spp.*	0 (0%)	3 (1%)	3 (1%)
Norovirus	0 (0%)	1 (1%)	2 (1%)
Pantoea agglomerans	0 (0%)	1 (1%)	2 (1%)
Pegivirus A/C	0 (0%)	2 (1%)	2 (1%)
Peptostreptococcus anaerobius	0 (0%)	1 (1%)	2 (1%)
Porphyromonas asacchrolytica	1 (1%)	0 (0%)	2 (1%)
Prevotella spp.	0 (0%)	18 (8%)	18 (8%)
Pseudomonas spp.*	7 (3%)	21 (10%)	28 (13%)
Rhodococcus hoagie	0 (0%)	1 (1%)	2 (1%)
Rothia mucilaginosa	0 (0%)	1 (1%)	2 (1%)
Rubella virus	0 (0%)	1 (1%)	2 (1%)
Saccharomyces cerevisiae	0 (0%)	1 (1%)	2 (1%)
Small circular virus family	0 (0%)	2 (1%)	2 (1%)
Snethia amnii	0 (0%)	1 (1%)	1 (1%)
Sphingobium yanoikuyae	0 (0%)	6 (3%)	6 (3%)
Staphylococcus epidermidis	0 (0%)	1 (1%)	2 (1%)
Stenotrophomonas rhizophila	1 (1%)	4 (2%)	5 (2%)
Streptococcus spp.*	7 (3%)	40 (18%)	47 (22%)
Tannerella spp.	0 (0%)	1 (1%)	2 (1%)
Tropheryma whipplei	0 (0%)	1 (1%)	2 (1%)
Veillonella species	11 (5%)	48 (22%)	59 (28%)

*Excluding established pathogens within this genus.

Supplementary Table 7. Comparison between RNA-seq and viral multiplex PCR

Patient ID	Virus detected by RNA-seq	Virus detected by RT-PCR
**1242**	Rhinovirus B	Rhinovirus/Enterovirus
**1424**	Human metapneumovirus	Human metapneumovirus
**1497**	Rhinovirus A	Rhinovirus/Enterovirus
**1630**	Rhinovirus A	Rhinovirus/Enterovirus
**1662**	Rhinovirus A	Rhinovirus/Enterovirus
**1689**	Rhinovirus A	Rhinovirus/Enterovirus
**1696**	Human metapneumovirus	Human metapneumovirus
**1790**	Human parainfluenza virus 3	Parainfluenza 3
**1913**	Rhinovirus A	Rhinovirus/Enterovirus
**1944**	Rhinovirus A	Rhinovirus/Enterovirus
**1908**	Rhinovirus C	Rhinovirus/Enterovirus
**1986**	Rhinovirus B	Rhinovirus/Enterovirus
**2044**	Rhinovirus A	Rhinovirus/Enterovirus
**2195**	Rhinovirus B	Rhinovirus/Enterovirus
**2305**	Influenza B virus	Influenza B
**2306**	Human parainfluenza virus 2	Parainfluenza 2
**2357**	Influenza A virus	Influenza A H3
**2321**	Influenza A virus + Rhinovirus C	Influenza A H3, Rhinovirus/Enterovirus
**2343**	Influenza A virus + Rhinovirus B	Influenza A H1, Rhinovirus/Enterovirus
**2413**	Rhinovirus A	Rhinovirus/Enterovirus

Supplementary Table 8. Comparison between RNA-seq and 16s rRNA gene sequencing for bacterial taxa detected by the rules-based model.

Patient ID	Genus identified by RNA-seq	Abundance Rank by 16S
1138	*Haemophilus*	1
1138	*Streptococcus*	2
1142	*Pseudomonas*	7
1165	*Veillonella*	3
1166	*Veillonella*	3
1166	*Pseudomonas*	28
1169	*Pseudomonas*	66
1175	*Pseudomonas*	63
1186	*Pseudomonas*	47
1194	*Pseudomonas*	34
1197	*Pseudomonas*	11
1200	*Pseudomonas*	16
1216	*Haemophilus*	1
1216	*Streptococcus*	2
1254	*Streptococcus*	3
1266	*Streptococcus*	4
1266	*Pseudomonas*	17
1289	*Haemophilus*	2
1290	*Streptococcus*	3
1292	*Streptococcus*	2
1325	*Veillonella*	3
1325	*Pseudomonas*	75
1328	*Lactobacillus*	3
1328	*Actinomyces*	8
1328	*Pseudomonas*	31
1341	*Sphinobium*	3
1341	*Pseudomonas*	107
1342	*Neisseria*	1
1363	*Pseudomonas*	26
1390	*Streptococcus*	1
1396	*Veillonella*	3
1396	*Pseudomonas*	39
1397	*Pseudomonas*	11
1416	*Haemophilus*	35
1416	*Neisseria*	39
1420	*Pseudomonas*	28
1424	*Streptococcus*	3
1424	*Veillonella*	4
1424	*Pseudomonas*	78
1426	*Streptococcus*	6
1426	*Haemophilus*	16
1426	*Lactobacillus*	81
1428	*Mycobacterium*	1
1438	*Prevotella*	1
1438	*Tannerella*	14
1442	*Pseudomonas*	71
1444	*Pseudomonas*	36
1446	*Haemophilus*	3
1477	*Haemophilus*	2
1477	*Sphinobium*	3
1477	*Pseudomonas*	56
1477	*Neisseria*	366

1499	*Sphinobium*	1
1549	*Pseudomonas*	65
1571	*Stenotrophomonas*	51
1586	*Veillonella*	2
1591	*Actinomyces*	3
1594	*Sphinobium*	1
1594	*Pseudomonas*	33
1598	*Prevotella*	1
1612	*Acinetobacter*	20
1618	*Veillonella*	5
1625	*Veillonella*	3
1628	*Streptococcus*	1
1651	*Haemophilus*	1
1665	*Veillonella*	2
1674	*Mycobacterium*	21
1690	*Haemophilus*	1
1714	*Veillonella*	4
1717	*Achromobacter*	1
1717	*Veillonella*	4
1720	*Prevotella*	1
1720	*Veillonella*	3
1728	*Streptococcus*	1
1747	*Veillonella*	3
1753	*Haemophilus*	4
1759	*Capnocytophaga*	4
1773	*Streptococcus*	1
1774	*Streptococcus*	1
1774	*Veillonella*	3
1776	*Streptococcus*	3
1776	*Pseudomonas*	8
1777	*Pseudomonas*	22
1779	*Actinomyces*	3
1784	*Sphinobium*	1
1784	*Pseudomonas*	7
1790	*Streptococcus*	2
1794	*Rothia*	3
1795	*Prevotella*	1
1795	*Veillonella*	3
1798	*Streptococcus*	2
1798	*Veillonella*	3
1799	*Pseudomonas*	4
1804	*Streptococcus*	2
1813	*Pseudomonas*	4
1816	*Haemophilus*	3
1827	*Prevotella*	1
1827	*Streptococcus*	2
1839	*Pseudomonas*	8
1845	*Stenotrophomonas*	3
1847	*Veillonella*	3
1851	*Sphinobium*	1
1858	*Pseudomonas*	1
1871	*Pseudomonas*	5
1872	*Streptococcus*	1
1884	*Pseudomonas*	3
1891	*Stenotrophomonas*	8
1896	*Haemophilus*	1
1900	*Pseudomonas*	3
1917	*Streptococcus*	3
1930	*Fusobacterium*	4
1930	*Actinomyces*	16
1935	*Streptococcus*	1
1935	*Veillonella*	6

1942	*Streptococcus*	1
1944	*Veillonella*	4
1945	*Streptococcus*	1
1965	*Streptococcus*	1
1965	*Actinomyces*	2
1965	*Veillonella*	5
1978	*Capnocytophaga*	5
1985	*Haemophilus*	1
1986	*Streptococcus*	1
1986	*Neisseria*	2
1987	*Streptococcus*	1
1987	*Haemophilus*	2
1987	*Mycoplasma*	3
1990	*Veillonella*	3
2001	*Veillonella*	3
2030	*Streptococcus*	1
2030	*Prevotella*	2
2030	*Lactobacillus*	4
2032	*Haemophilus*	1
2090	*Streptococcus*	1
2100	*Prevotella*	1
2100	*Veillonella*	2
2100	*Streptococcus*	3
2100	*Lactobacillus*	4
2102	*Veillonella*	3
2105	*Streptococcus*	2
2123	*Veillonella*	2
2134	*Streptococcus*	2
2135	*Streptococcus*	1
2140	*Haemophilus*	1
2142	*Veillonella*	2
2152	*Actinomyces*	2
2152	*Veillonella*	3
2154	*Veillonella*	2
2164	*Streptococcus*	2
2169	*Enterococcus*	5
2174	*Prevotella*	1
2174	*Streptococcus*	2
2174	*Veillonella*	3
2177	*Prevotella*	1
2179	*Prevotella*	1
2179	*Streptococcus*	2
2179	*Veillonella*	3
2179	*Anaerococcus*	4
2179	*Peptostreptococcus*	5
2179	*Leptotrichia*	6
2179	*Staphylococcus*	8
2179	*Fineoldia*	10
2180	*Streptococcus*	1
2188	*Veillonella*	2
2192	*Streptococcus*	1
2192	*Prevotella*	2
2194	*Veillonella*	4
2205	*Streptococcus*	4
2206	*Streptococcus*	3
2208	*Veillonella*	1
2212	*Streptococcus*	2
2216	*Streptococcus*	1
2216	*Veillonella*	2
2239	*Veillonella*	2
2241	*Mycoplasma*	8
2242	*Porphyromonas*	2

2257	*Veillonella*	2
2261	*Veillonella*	3
2316	*Actinomyces*	2
2316	*Leptotrichia*	4
2316	*Veillonella*	7
2316	*Campylobacter*	13
2321	*Haemophilus*	1
2323	*Rhodococcus*	364
2336	*Streptococcus*	3
2343	*Veillonella*	3
2352	*Veillonella*	2
2357	*Veillonella*	2
2361	*Veillonella*	3
2378	*Streptococcus*	2
2378	*Veillonella*	3
2413	*Haemophilus*	1
2432	*Prevotella*	1
2432	*Veillonella*	3
2442	*Streptococcus*	1
2444	*Veillonella*	3
2455	*Veillonella*	3
2460	*Veillonella*	3

Supplementary Table 9. Data and statistical calculations for CD4 count and pathogen detection associated with Figure 3. P values determined by Fisher's exact test. Legend: Adjusted p values based on Benjamini-Hochberg multiple test correction.

Pathogen	CD4 < 200	CD4 ≥ 200	Fisher p	Adjusted p
Anelloviridae	43	6	0.036	0.476
No *Anelloviridae*	123	45		
P. jirovecii	12	0	0.073	0.476
No *P. jirovecii*	154	51		
HHV-8	17	1	0.080	0.476
No *HHV-8*	149	50		
Streptococcus spp.	40	7	0.125	0.476
No *Streptococcus spp.*	126	44		
Pseudomonas spp.	18	10	0.149	0.476
No *Pseudomonas spp.*	148	41		
H. influenzae	18	2	0.172	0.476
No *H. influenzae*	148	49		
M. tuberculosis	30	5	0.195	0.476
No *M. tuberculosis*	136	46		
CMV	21	3	0.211	0.476
No *CMV*	145	48		
Rhinovirus A,B,C	29	5	0.270	0.540
No *Rhinovirus A,B,C*	137	46		
S. pneumoniae	4	3	0.359	0.625
No *S. pneumoniae*	162	48		
Prevotella spp.	12	6	0.382	0.625
No *Prevotella spp.*	154	45		
P. aeruginosa	8	4	0.483	0.686
No *P. aeruginosa*	158	47		
EBV	55	14	0.495	0.686
No *EBV*	111	37		
Veillonella spp.	47	12	0.591	0.753
No *Veillonella spp.*	119	39		
S. yanoikuyae	4	2	0.627	0.753
No *S. yanoikuyae*	162	49		
K. pneumoniae	6	1	1.000	1.000
No *K. pneumoniae*	160	50		
Influenza.A	5	1	1.000	1.000
No *Influenza.A*	161	50		
Actinomyces spp.	8	2	1.000	1.000
No *Actinomyces spp.*	158	49		

Supplementary Table 10. Data and statistical calculations for mortality and pathogen detection associated with Figure 4. P values determined by Fisher's exact test. Legend: Adjusted p values based on Benjamini-Hochberg multiple test correction.

Pathogen	Not alive	Alive	Fisher p	Adjusted p
HHV-8	11	4	3.90×10^{-5}	7.38×10^{-4}
No HHV-8	36	143		
K. pneumoniae	4	3	0.060	0.376
No *K. pneumoniae*	43	144		
P. aeruginosa	0	11	0.069	0.376
No *P. aeruginosa*	47	136		
HSV-1	3	2	0.093	0.376
No HSV-1	44	145		
Actinomyces spp.	4	4	0.099	0.376
No *Actinomyces spp.*	43	143		
Rhinovirus A,B,C	10	21	0.260	0.715
No *Rhinovirus A,B,C*	37	126		
EBV	18	44	0.287	0.715
No EBV	29	103		
S. pneumoniae	0	5	0.338	0.715
No *S. pneumoniae*	47	142		
S. yanoikuyae	0	6	0.339	0.715
No *S. yanoikuyae*	47	141		
M. tuberculosis	10	22	0.366	0.715
No *M. tuberculosis*	37	125		
Veillonella spp.	15	37	0.449	0.776
No *Veillonella spp.*	32	110		
Prevotella spp.	2	12	0.524	0.801
No *Prevotella spp.*	45	135		
Anelloviridae	12	31	0.548	0.801
No *Anelloviridae*	35	116		
Pseudomonas spp.	5	21	0.629	0.853
No *Pseudomonas spp.*	42	126		
CMV	4	16	0.787	0.997
No *CMV*	43	131		
Streptococcus spp.	11	32	0.841	0.999
No *Streptococcus spp.*	36	115		
P. jirovecii	2	9	1.000	1.000
No *P. jirovecii*	45	138		
Influenza A	1	4	1.000	1.000
No *Influenza A*	46	143		
S. rhizophila	1	4	1.000	1.000
No S. rhizophila	46	143		

Supplementary Table 11. Lower respiratory tract antimicrobial resistance genes identified in each patient. Tet = tetacycline, AGly = aminoglycoside, Bla = beta-lactamase, MLS = macrolide, lincosomide, streptogramin, Sul = sulfa, Phe = phenicol, Flq = fluoroquinolone, Gly = glycopeptide, Tmt = trimethoprim.

ID	Gene	Class
**1884**	*SulI*	Sul
**1813**	*StrA*	AGly
**1813**	*SulI*	Sul
**1829**	*StrA*	AGly
**1829**	*ErmX*	MLS
**1829**	*QnrB*	Flq
**1829**	*AadA*	AGly
**1829**	*StrB*	AGly
**1829**	*TetG*	Tet
**1829**	*SulI*	Sul
**1790**	*CfxA*	Bla
**1790**	*TetM*	Tet
**1790**	*TetO*	Tet
**1790**	*TetQ*	Tet
**1416**	*OqxB*	Flq
**1416**	*TetC*	Tet
**1598**	*TetM*	Tet
**1598**	*MsrD*	MLS
**1598**	*CTX-M-1*	Bla-ESBL
**1243**	*OqxB*	Flq
**1243**	*TetC*	Tet
**1138**	*StrA*	AGly
**1138**	*OqxB*	Flq
**1138**	*CatQ*	Phe
**1138**	*TetB*	Tet
**1138**	*PBP1b*	Bla
**1138**	*Aph3''Ia*	AGly
**1138**	*SulII*	Sul
**1138**	*CatA2*	Phe
**1397**	*SulI*	Sul
**1397**	*AacAad*	AGly
**1289**	*TetC*	Tet
**1200**	*VanT*	Gly
**1541**	*OqxB*	Flq
**1592**	*StrB*	AGly
**1592**	*PBP1b*	Bla
**1592**	*PBP1a*	Bla
**1592**	*CTX-M-1*	Bla-ESBL
**1142**	*TetC*	Tet
**1254**	*PBP1b*	Bla
**1254**	*Aph3''Ia*	AGly
**1254**	*TlrC2*	MLS
**1216**	*CatA2*	Phe
**1396**	*OqxB*	Flq
**1446**	*ErmB*	MLS
**1446**	*PBP1a*	Bla
**1242**	*StrB*	AGly
**1242**	*StrA*	AGly
**1242**	*SulII*	Sul

**1342**	*PBP1a*	Bla
**1342**	*TetM*	Tet
**1571**	*AadD*	AGly
**1571**	*Aph4-Ia*	AGly
**1571**	*TetB*	Tet
**1571**	*SulI*	Sul
**1571**	*CatA9*	Phe
**1175**	*CatA9*	Phe
**1290**	*PBP1b*	Bla
**1290**	*TetM*	Tet
**1290**	*TetQ*	Tet
**1442**	*OleC*	MLS
**1442**	*TetC*	Tet
**1424**	*OqxB*	Flq
**1424**	*AMPH*	Bla
**1328**	*OqxB*	Flq
**1328**	*AMPH*	Bla
**1549**	*MsrD*	MLS
**1549**	*TetC*	Tet
**1390**	*VanT*	Gly
**1438**	*TetW*	Tet
**1438**	*OqxB*	Flq
**1186**	*MsrE*	MLS
**1292**	*PBP1b*	Bla
**1428**	*TetC*	Tet
**1428**	*AmpC1*	Bla-ESBL
**1341**	*CfxA*	Bla
**1166**	*CTX-M-1*	Bla-ESBL
**1555**	*VanT*	Gly
**1205**	*CTX-M-1*	Bla-ESBL
**1205**	*Aac3-I*	AGly
**1205**	*HugA2*	Bla
**1205**	*SulII*	Sul
**1205**	*Aac3-Ib*	AGly
**1488**	*CfxA*	Bla
**1488**	*TetM*	Tet
**1747**	*CfxA*	Bla
**1747**	*TetW*	Tet
**1747**	*PBP1a*	Bla
**1747**	*MefA*	MLS
**1803**	*TlrC2*	MLS
**1803**	*TetM*	Tet
**1803**	*TetW*	Tet
**1803**	*TetO*	Tet
**1803**	*MsrD*	MLS
**1803**	*TetS*	Tet
**1803**	*MefA*	MLS
**1803**	*TetQ*	Tet
**1803**	*CfxA*	Bla
**1803**	*PBP1b*	Bla
**1759**	*TetM*	Tet
**1816**	*StrA*	AGly
**1816**	*TetB*	Tet
**1753**	*TetC*	Tet
**1899**	*Aac3-IIa*	AGly
**1899**	*QepA*	Flq
**1899**	*TetB*	Tet
**1899**	*TetM*	Tet
**1899**	*CTX-M-1*	Bla-ESBL
**1899**	*OXY2*	Bla
**1899**	*CatA9*	Phe
**1899**	*DfrA*	Tmt

**1899**	*PBP1a*	Bla
**1899**	*Aac3-I*	AGly
**1899**	*Aac3-Ib*	AGly
**1899**	*TetC*	Tet
**1899**	*CatBx*	Phe
**1899**	*PBP1b*	Bla
**1899**	*RAHN-1*	Bla
**1899**	*Aph2"I*	AGly
**1899**	*SulII*	Sul
**1899**	*CatA1*	Phe
**1829**	*StrA*	AGly
**1829**	*ErmX*	MLS
**1829**	*QnrB*	Flq
**1829**	*AadA*	AGly
**1829**	*StrB*	AGly
**1829**	*TetG*	Tet
**1829**	*SulI*	Sul
**1790**	*CfxA*	Bla
**1790**	*TetM*	Tet
**1790**	*TetO*	Tet
**1790**	*TetQ*	Tet
**1773**	*ErmE*	MLS
**1773**	*CatA1*	Phe
**1857**	*SulI*	Sul
**1630**	*TetA*	Tet
**1630**	*Aac3-IIa*	AGly
**1630**	*QnrB*	Flq
**1630**	*CTX-M-8*	Bla-ESBL
**1630**	*AacAad*	AGly
**1630**	*CTX-M-1*	Bla
**1630**	*OXY6*	Bla
**1630**	*StrA*	AGly
**1630**	*CatBx*	Phe
**1630**	*Aac3-I*	AGly
**1630**	*Aac3-Ib*	AGly
**1630**	*AadA*	AGly
**1630**	*StrB*	AGly
**1630**	*RAHN-1*	Bla
**1630**	*SED-1*	Bla
**1630**	*SulII*	Sul
**1630**	*OXA-1*	Bla
**1630**	*CatA1*	Phe
**1779**	*TetM*	Tet
**1779**	*ErmX*	MLS
**1779**	*MsrD*	MLS
**1779**	*ErmB*	MLS
**1779**	*MefA*	MLS
**1779**	*SPU*	Bla
**1779**	*CfxA*	Bla
**1884**	*SulI*	Sul
**1720**	*StrA*	AGly
**1720**	*FloR*	Phe
**1720**	*TetC*	Tet
**1872**	*MefA*	MLS
**1872**	*MsrD*	MLS
**1858**	*SulI*	Sul
**1804**	*TetC*	Tet
**1612**	*TlrC2*	MLS
**1612**	*Cmr*	Phe
**1612**	*TetC*	Tet
**1612**	*SHV-OKP-LEN*	Bla-ESBL
**1612**	*Aph3"Ia*	AGly

**1651**	*StrA*	AGly
**1651**	*CatBx*	Phe
**1651**	*TetB*	Tet
**1651**	*StrB*	AGly
**1651**	*Aph3''Ia*	AGly
**1651**	*SulII*	Sul
**1651**	*CatA2*	Phe
**1813**	*StrA*	AGly
**1813**	*SulI*	Sul
**1891**	*LnuA*	MLS
**1662**	*Aac3-IIa*	AGly
**1662**	*AacAad*	AGly
**1662**	*Aac3-Ib*	AGly
**1776**	*StrA*	AGly
**1784**	*StrA*	AGly
**1665**	*ErmB*	MLS
**1665**	*TetM*	Tet
**1777**	*SulI*	Sul
**1986**	*TetM*	Tet
**1986**	*Tet-32*	Tet
**1986**	*MsrD*	MLS
**1986**	*StrA*	AGly
**1986**	*PBP1a*	Bla
**1986**	*StrB*	AGly
**1986**	*Aph3''Ia*	AGly
**1986**	*SulII*	Sul
**1986**	*BRO*	Bla
**1864**	*MsrD*	MLS
**1864**	*TetC*	Tet
**1655**	*TetC*	Tet
**1655**	*LAP*	Bla
**1714**	*StrB*	AGly
**1714**	*ErmB*	MLS
**1714**	*CatQ*	Phe
**1714**	*ErmX*	MLS
**1851**	*SulI*	Sul
**1867**	*OXA-63*	Bla-ESBL
**1625**	*SulI*	Sul
**1618**	*Aac3-IIa*	AGly
**1618**	*QnrB*	Flq
**1618**	*TetQ*	Tet
**1618**	*SulII*	Sul
**1618**	*DfrA5*	Tmt
**1896**	*MsrD*	MLS
**1896**	*TetO*	Tet
**1896**	*TetM*	Tet
**1896**	*StrA*	AGly
**1896**	*OXA-63*	Bla-ESBL
**1896**	*Aph3''Ia*	AGly
**1896**	*SulII*	Sul
**1696**	*AacAad*	AGly
**1696**	*Aac3-Ib*	AGly
**1821**	*TetM*	Tet
**1821**	*ErmX*	MLS
**1821**	*TetL*	Tet
**1821**	*PBP1a*	Bla
**1821**	*VanL*	Gly
**1821**	*TetQ*	Tet
**1821**	*PBP1b*	Bla
**1821**	*Aph3''Ia*	AGly
**1821**	*SulII*	Sul
**1601**	*AMPH*	Bla

**1601**	*LAP*	Bla
**1601**	*TetC*	Tet
**1905**	*VanT*	Gly
**1827**	*Aph2"I*	AGly
**1798**	*TetQ*	Tet
**1728**	*TetM*	Tet
**1728**	*PBP1b*	Bla
**1728**	*MsrD*	MLS
**1728**	*CTX-M-1*	Bla-ESBL
**1728**	*StrA*	AGly
**1728**	*OqxB*	Flq
**1728**	*MefA*	MLS
**1728**	*Aac3-Ib*	AGly
**1728**	*StrB*	AGly
**1728**	*TetQ*	Tet
**1728**	*CfxA*	Bla
**1728**	*SulI*	Sul
**1728**	*SulII*	Sul
**1728**	*DfrA5*	Tmt

Supplementary Table 12. Incidence of *Pneumocystis jirovecii* and receipt of TMP-SMX prophylaxis (ppx).

	+ PJP	- PJP
**+ TMP-SMX ppx**	2	120
**- TMP-SMX ppx**	10	85

Supplementary Table 13. Trimethoprim-sulfamethoxazole (TMP-SMX) antimicrobial resistance (AMR) genes detected and receipt of TMP-SMX prophylaxis (ppx). A) 2 x 2 matrix of TMP-SMX ppx versus AMR genes detected. B) List of TMP and SMX AMR genes detected.

(A)

	+ AMR genes	- AMR genes
**+ TMP-SMX ppx**	14	108
**- TMP-SMX ppx**	7	88

(B)

Study ID	TMP-SMX ppx	Gene
**1138**	Yes	*SulII*
**1205**	Yes	*SulII*
**1242**	Yes	*SulII*
**1625**	Yes	*SulI*
**1630**	Yes	*SulII*
**1651**	Yes	*SulII*
**1728**	Yes	*SulI,DfrA5*
**1777**	Yes	*SulI*
**1813**	Yes	*SulI*
**1821**	Yes	*SulII*
**1829**	Yes	*SulI*
**1851**	Yes	*SulI*
**1858**	Yes	*SulI*
**1986**	Yes	*SulII*
**1397**	No	*SulI*
**1571**	No	*SulI*
**1618**	No	*SulII, DfrA5*
**1857**	No	*SulI*
**1884**	No	*SulI*
**1896**	No	*SulII*
**1899**	No	*SulII, DfrA*

Supplementary Table 14. Patient enrollment details.

**Enrollment details**	**n**
Enrolled	636
Underwent bronchoscopy	223
Unsuccessful bronchoscopy /very low or no return	6
Available samples for analysis	217
Eligible but did not undergo bronchoscopy	413
**Reasons for not doing bronchoscopy**	
Patient died	39
Patient too ill	34
Patient refused	34
Patient left against medical advice	11
Patient discharged	20
Patient direct AFB smear positive	41
Physician declined, alternate diagnosis	100
Patient in multi-drug resistant TB risk-group	44
Other	90
**Specific other reasons**	
Bronchoscopy procedure on hold	86
Disseminated TB	2
Started on TB treatment	1
Altered mental status	1

Supplementary Sources Cited

1 Iwai S, Huang D, Fong S, *et al.* The lung microbiome of Ugandan HIV-infected pneumonia patients is compositionally and functionally distinct from that of San Franciscan patients. *PLoS ONE* 2014; **9**: e95726.

2 Shenoy MK, Iwai S, Lin DL, *et al.* Immune Response and Mortality Risk Relate to Distinct Lung Microbiomes in Patients with HIV and Pneumonia. *Am J Respir Crit Care Med* 2017; **195**: 104–14.

3 Langelier C, Kalantar KL, Moazed F, *et al.* Integrating host response and unbiased microbe detection for lower respiratory tract infection diagnosis in critically ill adults. *Proc Natl Acad Sci USA* 2018; : 201809700.

4 Kalantar, Katrina. IDseq – An Open Source Cloud-based Pipeline and Analysis Service for Metagenomic Pathogen Detection and Monitoring. .

5 Dickson RP, Erb-Downward JR, Freeman CM, *et al.* Bacterial Topography of the Healthy Human Lower Respiratory Tract. *mBio* 2017; **8**: e02287-16.

6 Cruciani M, Marcati P, Malena M, Bosco O, Serpelloni G, Mengoli C. Meta-analysis of diagnostic procedures for Pneumocystis carinii pneumonia in HIV-1-infected patients. *Eur Respir J* 2002; **20**: 982–9.

7 Iwai S, Fei M, Huang D, *et al.* Oral and Airway Microbiota in HIV-Infected Pneumonia Patients. *Journal of Clinical Microbiology* 2012; **50**: 2995–3002.

8 Dobin A, Davis CA, Schlesinger F, Drenkow J, Zaleski C, Jha S. STAR: ultrafast universal RNA-seq aligner. *Bioinformatics* 2013; **29**. DOI:10.1093/bioinformatics/bts635.

9 Ruby JG, Bellare P, Derisi JL. PRICE: software for the targeted assembly of components of (Meta) genomic sequence data. *G3 (Bethesda)* 2013; **3**: 865–80.

10 Langmead B, Salzberg SL. Fast gapped-read alignment with Bowtie 2. *Nat Methods* 2012; **9**. DOI:10.1038/nmeth.1923.

11 Jain S, Self WH, Wunderink RG, *et al.* Community-Acquired Pneumonia Requiring Hospitalization among U.S. Adults. *N Engl J Med* 2015; **373**: 415–27.

12 Magill SS, O'Leary E, Janelle SJ, *et al.* Changes in Prevalence of Health Care–Associated Infections in U.S. Hospitals. *New England Journal of Medicine* 2018; **379**: 1732–44.

13 Mandell LA, Wunderink RG, Anzueto A, *et al.* Infectious Diseases Society of America/American Thoracic Society consensus guidelines on the management of community-acquired pneumonia in adults. *Clin Infect Dis* 2007; **44 Suppl 2**: S27-72.

14 Fishman JA. Infection in Organ Transplantation. *Am J Transplant* 2017; **17**: 856–79.

15 Kalil AC, Metersky ML, Klompas M, *et al.* Management of Adults With Hospital-acquired and Ventilator-associated Pneumonia: 2016 Clinical Practice Guidelines by the Infectious

Diseases Society of America and the American Thoracic Society. *Clin Infect Dis* 2016; **63**: e61–111.

16 Zinter MS, Dvorak CC, Mayday MY, *et al.* Pulmonary Metagenomic Sequencing Suggests Missed Infections in Immunocompromised Children. *Clinical Infectious Diseases* 2019; **68**: 1847–55.

17 Langelier C, Zinter MS, Kalantar K, *et al.* Metagenomic Sequencing Detects Respiratory Pathogens in Hematopoietic Cellular Transplant Patients. *Am J Respir Crit Care Med* 2017; published online July 7. DOI:10.1164/rccm.201706-1097LE.

18 Pine PS, Munro SA, Parsons JR, *et al.* Evaluation of the External RNA Controls Consortium (ERCC) reference material using a modified Latin square design. *BMC Biotechnology* 2016; **16**. DOI:10.1186/s12896-016-0281-x.

19 The External RNA Controls Consortium. The External RNA Controls Consortium: a progress report. *Nat Methods* 2005; **2**: 731–4.

20 Wilson MR, O’Donovan BD, Gelfand JM, *et al.* Chronic Meningitis Investigated via Metagenomic Next-Generation Sequencing. *JAMA Neurol* 2018; **75**: 947–55.

21 Inouye M, Dashnow H, Raven L-A, *et al.* SRST2: Rapid genomic surveillance for public health and hospital microbiology labs. *Genome Med* 2014; **6**: 90.
